# Interoceptive accuracy and bias in somatic symptom disorder, illness anxiety disorder, and functional syndromes: A systematic review and meta-analysis

**DOI:** 10.1371/journal.pone.0271717

**Published:** 2022-08-18

**Authors:** Carolin Wolters, Alexander L. Gerlach, Anna Pohl

**Affiliations:** Department of Psychology, Institute of Clinical Psychology and Psychotherapy, University of Cologne, Cologne, Germany; Anglia Ruskin University, UNITED KINGDOM

## Abstract

Somatic symptom disorder, illness anxiety disorder, and functional syndromes are characterized by burdensome preoccupation with somatic symptoms. Etiological models propose either increased interoceptive accuracy through hypervigilance to the body, or decreased and biased interoception through top-down predictions about sensory events. This systematic review and meta-analysis summarizes findings of 68 studies examining interoceptive accuracy and 8 studies examining response biases in clinical or non-clinical groups. Analyses yielded a medium population effect size for decreased interoceptive accuracy in functional syndromes, but no observable effect in somatic symptom disorder and illness anxiety disorder. The overall effect size was highly heterogeneous. Regarding response bias, there was a small significant effect in somatic symptom disorder and illness anxiety disorder. Our findings strengthen the notion of top-down factors that result in biased rather than accurate perception of body signals in somatic symptom disorder and illness anxiety disorder.

## Introduction

### Interoception in the etiology of somatic symptom and related disorders

Somatic symptom and related disorders are characterized by distressing somatic symptoms and their interference with daily life. Thoughts, affect and behavior concerning the symptoms are unreasonable and result in extensive personal burden such as excessive preoccupation and societal costs like increased medical utilization [1–5]. Unfortunately, psychotherapy for these disorders has merely moderate effects [for cognitive behavioral treatment, see 6, 7]. Enhancing knowledge about pathological mechanisms is therefore essential [8]. Interoception, as the processing, integration, and interpretation of bodily signals, is a promising candidate in this regard [9]. Some etiological approaches propose increased, but others decreased and biased sensitivity for interoceptive signals [8].

Based on findings of higher levels of arousal and perceptual sensitivity [10] and lower pain tolerance in hypochondriasis [11, 12] and chronic pain [13], it was assumed that illness anxiety and somatic symptom burden result from misinterpreted perception of *actual* physiological changes. According to this theory, affected individuals perceive even the slightest fluctuations in physiological signals because of heightened attention to the body. Body signals are then misinterpreted as pathological, uncomfortable, and stressful, which in turn reinforces hypervigilant attention to the body. Thereby symptoms are maintained [14, 15]. In these etiological frameworks, individuals with high somatic symptom burden are assumed to exhibit *higher* interoceptive accuracy (IAcc). Contrary to this notion, the predictive coding perspective proposes that symptoms unfold increasingly *independent* of *actual* physiological changes over the course of somatic symptom and related disorders [16]. According to this view, somatic symptoms result from individual prediction models, rather than the processing of somatosensory input per se. To construct a perception, the brain matches predictions with somatosensory input. Although predictions need not be correct, they can still be given more weight when physiological stimuli are ambiguous [17]. In other words, somatic symptoms might be perceived independently of psychophysiological changes, when they are predicted as the most likely input. Physical changes are also more likely experienced as signs of an illness when the individual model predicts that their most likely cause is an illness. The less detailed sensory signals are processed, the more likely predictions about the presence of symptoms and disease will lead to the perception of symptoms. Attention, negative affect and gender are assumed to moderate these processes [16]. Therefore, *reduced* and *biased* interoception is hypothesized in the context of somatic symptom disorders [16]. Biased perception presumably follows a “better safe than sorry” approach, where patients affirm bodily symptoms more readily and answer more liberally in ambiguous situations [18]. In other words, threat-related predictions dominate perceptual processes and lead to illusory sensations, an information processing style that might be common in psychopathology [16].

However, empirical findings on IAcc are heterogeneous, showing increased [19, 20], unchanged [21–24], and diminished IAcc [25–28] in somatic symptom disorders. Concerning interoceptive biases, a number of studies documented a more liberal response bias (RB) in people with somatoform symptoms [19, 29–31].

#### Aims of this article

In the light of recent advances in interoception research, our aim was to assess both IAcc and RB in different diagnostic categories of somatic symptom and related disorders in a comprehensive systematic review and meta-analysis. We give an overview of the methods used in prior studies investigating IAcc and RB and conduct statistical analyses to calculate population effect sizes, identify potential moderators and check for publication bias. This overview was generated without preregistration or external protocol.

## Methods

### Diagnostic conceptualization

Conceptualization of somatic symptom and related disorders is difficult, inconsistent [32, 33], and recently underwent remarkable changes at least partially motivated by the lack of recognition of “somatoform disorders” in primary care and other medical settings [32]. Concerning somatic symptom disorder, the fifth edition of the *Diagnostic and Statistical Manual of Mental Disorders* [DSM-5, 5] introduced symptom-related abnormal behaviors, thoughts, and feelings as diagnostic criteria, while lack of medical explanation was dropped. Psychological mechanisms also play a role in the maintenance of functional syndromes [34, 35]. Moreover, patients suffering from functional syndromes often report symptoms outside of their respective symptom complex [36–39]. Consequently, quite a few authors emphasize the overlap between different somatic symptom and functional disorders [e.g. 37, 40, 41].

However, DSM-5 has been criticized for bringing together heterogeneous clinical conditions, as it is yet unclear whether medically explained and unexplained somatic symptoms involve similar mechanisms [33]. As research following DSM-5 criteria for somatic symptom and related disorders is limited, current knowledge relates to previous DSM conceptualizations that highlighted the absence of satisfactory medical explanations of bodily symptoms rather than psychological mechanisms [42].

Against this background, we chose to aggregate diverse concepts of somatic symptom and related disorders, using the DSM-5 label as an umbrella term. We included previous and current psychological classifications as well as functional syndromes with unknown biomedical etiology. This inclusive approach prevents a loss of power and allows unfolding both mutual and distinct characteristics of these clinical pictures with regard to IAcc.

### Information sources and search strategies

We searched *PsychInfo*, *Medline*, *Web of Science*, and *ProQuest Dissertations & Theses* databases on June 24^th^, 2016, using keywords such as “interocept*”, “propriocept*”, and “health anxiety”, “somatoform”, “fibromyalgia” as well as other terms for somatoform and functional syndromes (see S1 Table). The search was updated on November 3^rd^, 2020, using the same search terms. We scanned bibliographical references of the included studies and checked for studies that cited the included studies published before 2000 using cited reference searches on *Web of Science*. If relevant statistical outcomes were not reported in an article, the corresponding author was contacted up to three times and asked to provide us with the data.

### Study selection

Two of the authors assessed the eligibility of studies. The third author was consulted in case of disagreement, and discrepancies were solved by consensus. All authors agreed on the final inclusion of studies (*k* = 69).

#### General eligibility criteria

Articles in English or German language were considered for inclusion. We included studies comparing an experimental group diagnosed with somatic symptom disorder, illness anxiety disorder, or functional syndromes with a gender-matched healthy control population. We also included studies that assessed correlations between symptoms of these categories and IAcc/RB outcomes in non-clinical samples. We did not set any other restriction concerning study design.

#### Participant characteristics

We only included studies testing adult samples. In clinical samples, the experimental group had to be diagnosed based on either a standardized classification system such as the DSM or ICD, disorder-specific criteria, or by evaluation of an expert in the field. Based on comprehensive research on specific diagnoses, we included only functional syndromes for which no sufficient organic etiology is known and verified (i.e., cervical dystonia, cervicogenic headache, functional vestibular symptoms). Physiological abnormalities were accepted if they had not preceded symptom onset, such as muscle tension in headache. Samples with potential or confirmed underlying organic causes for functional syndromes were excluded (e.g. [43, 44]). Non-clinical samples were included when a correlation between interoceptive measures and an established symptom questionnaire was provided.

#### Task characteristics

While initial concepts of interoception solely referred to visceral afferent information [45], recent definitions include stimuli arising anywhere in the body, such as the skin and proprioceptive functions [46, 47]. This definition redundantizes the separation between “interoception” vs. “exteroception”, arguing that not the stimulus origin but the perception in the central nervous system are decisive [48].

We considered tasks measuring different interoceptive domains that result in a quantifiable measure of IAcc: the perception of visceral, skin conductance, and muscle information, perception of body position, and perception of tactile stimulation on skin. We did not include tasks prone to interference by abilities other than IAcc, such as proprioceptive tasks allowing visual feedback [e.g. in 49]. Tasks that used symptom induction [as in 50] or explicit impediment of IAcc [e.g. using additional weights in a proprioceptive task in 51, 52] were excluded.

#### Outcome characteristics

The outcome for IAcc had to be a quantifiable match between an objectively measured physiological signal and its perception. For example, correlation scores of self-report and physiological measures, threshold scores referring to the lowest intensity at which an individual perceives a certain stimulus, reversed error scores, or values based on signal detection theory were included. In the somatic signal detection task, stimuli selection is based on a thresholding procedure that leads to similar *d’* scores across participants. Therefore, *d’* is not a meaningful score in this task when comparing accuracy between groups. Here, thresholding scores were used as measures of accuracy.

RB outcomes were considered for separate analyses. We included values derived from signal detection theory such as *c*, and *β*, a nonparametric measure of RB [53].

### Data extraction and analysis

Two of the authors independently extracted the following study data: sample size, task, diagnosis, symptom-specific questionnaires, and primary outcome measures. Relevant outcome parameters for IAcc and RB were abstracted to an Excel sheet (Version 16.43) and analyzed using the metafor package [54] for R (Version 3.3.1).

### Calculation of effect sizes

We computed effect sizes expressed as correlation coefficients *r* from outcome measures reported in the included studies (means and sample sizes, *z*-scores and sample sizes, or *F*-scores and their denominator degrees of freedom). Coefficients were computed such that negative values represent lower IAcc in the experimental group than the control group. Correlation coefficients were computed as outlined in [55] and [56]. For RB, effect sizes were calculated such that negative values indicated a more liberal response style in the experimental group.

For studies providing multiple results, we followed Rosenthal’s [57] recommendation in averaging *z*-transformed *r’*s such that each study would contribute a single effect size estimate to the overall analysis. If multiple study outcomes could be assigned to superordinate categories within that study, lower order outcomes (e.g., different task outcomes) were averaged before averaging higher order outcomes (e.g., different experimental groups). Quantities of averaged outcomes are shown in S2 Table. Two studies reported multiple outcomes for non-clinical illness anxiety [29, 58]. Here, we chose to only extract data of the WI [59], as this represents a widely accepted measure of non-clinical illness anxiety and thus allows comparability across studies.

### Method of meta-analysis

We calculated random-effects models with standardized correlations (*r*_*z*_) according to Hedges and Vevea [60]. Their method provides *Q* statistics of homogeneity of effect sizes. This test has low power when few studies are included [61], but is too sensitive when the number of included studies is high [62]. As additional measures of heterogeneity, *H*^*2*^ and *I*^*2*^ according to Higgins and Thompson [63] are reported. For *H*^*2*^ values exceeding 1.5, considerable caution is advised [63]. Although official criteria for the interpretation of *I*^2^ are lacking, values ≥ 75% are considered high [62].

We conducted four separate moderator analyses using mixed models [64] with the following categorical moderators: 1) sample (clinical, non-clinical), 2) diagnosis (functional syndromes, somatic symptom disorder, illness anxiety disorder), 3) interoceptive domain (visceral/muscle perception, tactile, proprioceptive), and 4) task type (signal detection, thresholding procedure, heartbeat mental tracking, position sense, rubber hand illusion, and correlational tasks). Subgroup analyses were calculated for significant moderators.

### Risk of bias evaluation

Standardized checklists for risk of bias are provided for epidemiological [65] and intervention studies [66]. We based our risk of bias evaluation on Di Lernia and colleagues [67]. Assessment criteria included sampling and matching procedures, citation of assessment protocols, and handling of missing data. We did not check for IAcc interference factors, as these vary strongly depending on bodily domain and task type. Furthermore, there are no scientific standards for most domains (with exception of the cardiovascular body domain). Two of the authors rated the criteria and solved discrepancies by consensus.

### Publication bias assessment

The trim and fill method according to Duval and Tweedie [68] was applied to a funnel plot of *z*-transformed correlations and standard errors. Egger’s regression test for funnel plot asymmetry [69], Rosenthal’s fail safe *N* [70], and Begg and Mazumdar’s rank correlation test [71] are also reported. In addition, Vevea and Woods’ [72] sensitivity analysis was conducted to quantify the likely effect of publication bias, using the R code by Field and Gillet [64].

## Results

**Fig 1:**
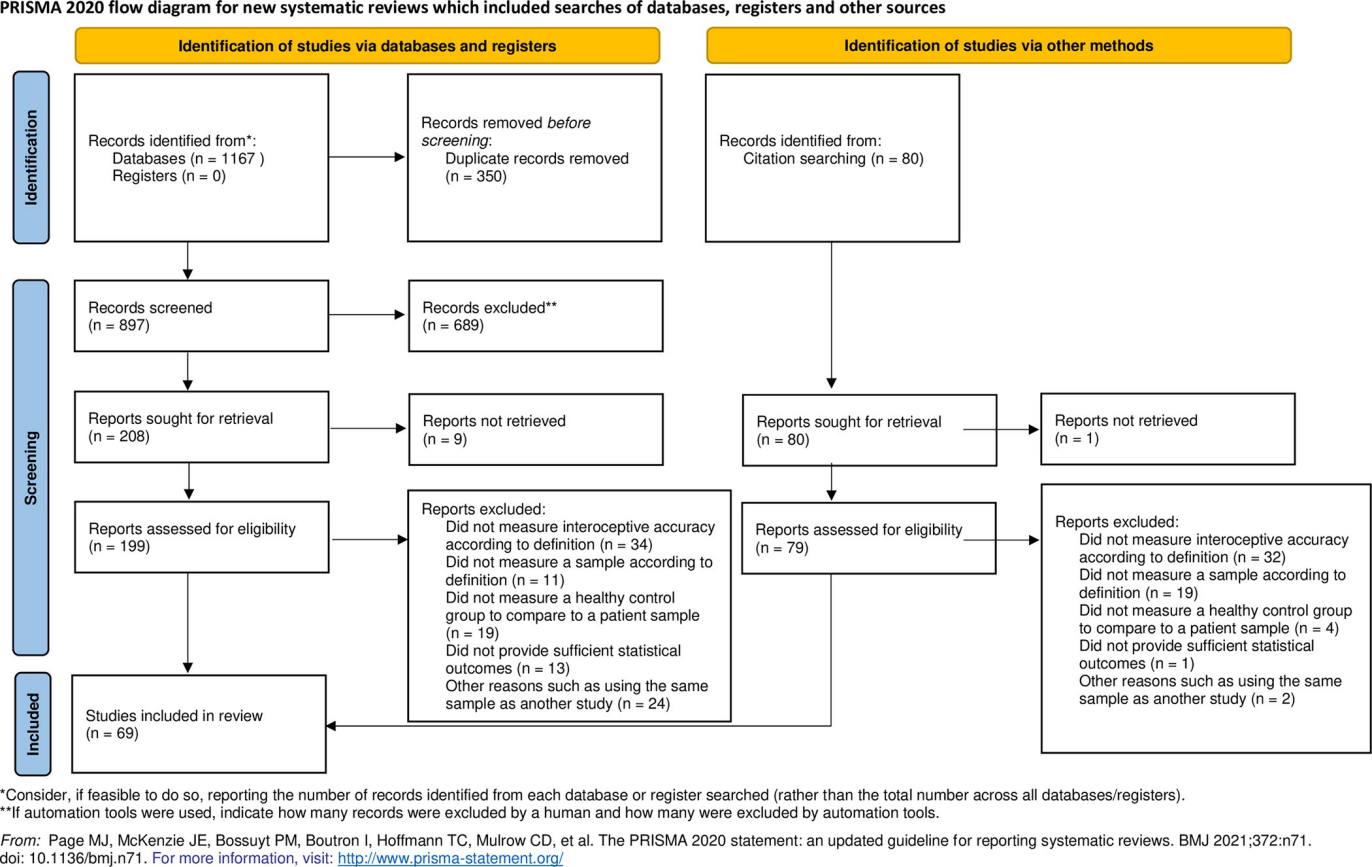
PRISMA flow chart of study inclusion.

Fig 1 shows the search process in a flow chart according to the PRISMA statement [73].

### Study characteristics

Characteristics of the studies varied considerably (see S2 Table for a detailed overview). Two articles were written in German, the remaining articles were written in English.

#### Samples

The majority of included studies assessed clinical samples (*k* = 54 vs. *k* = 15). Most studies (*k* = 43) examined functional syndromes such as fibromyalgia, dystonia, or neck pain, using a variety of diagnostic instruments (see S2 Table). Somatic symptom disorder was assessed in 23 studies and illness anxiety disorder in 6 studies. Clinical samples were most commonly diagnosed using the ICD-10 [74] and DSM-IV [75]. Questionnaires for non-clinical somatic symptoms and illness anxiety included the Patient Health Questionnaire [76], the Checklist for Symptoms in Daily Life [77], and the Whiteley Index [59].

#### IAcc and RB tasks

Interoception was assessed in the cardiovascular, muscular, proprioceptive, respiratory, electrodermal, and tactile body domain with a great variety of task types. We classified the tasks into the following six task types: heartbeat mental tracking tasks, signal detection tasks, thresholding procedures, position sense tasks, the rubber hand illusion paradigm, and correlational tasks. A description of task types can be found in S3 Table.

### Results of data analyses

#### Effects of IAcc

Effect sizes of all studies are shown in Table 1. The mean effect size based on Hedges and Vevea’s random-effects model for IAcc was *r*_*z*_ = -.196, a small effect according to Cohen [78], with a 95% confidence interval of *r*_*z*_ = -.282 (lower) and *r*_*z*_ = -.110 (upper). The associated *z*-score was highly significant (*z* = 4.46, *p* < .001). Estimated between-study variance was τ^*2*^ = .106, *I*^*2*^ = 85.70%, *H*^*2*^ = 6.99. *Q* test of homogeneity of effect sizes was significant *Q* = 312.74, *df* = 67, *p* < .001). The calculations suggest considerable variations in the effect sizes.

**Table 1:** Effect sizes of interoceptive accuracy (unstandardized *r*) for included studies.

First author (reference)	Diagnosis/symptoms of experimental group	Task	*N*	IAcc
	Somatic symptom disorders/illness anxiety disorders			
Barsky [79]	Hypochondriasis	Heartbeat discrimination task	105	.0851
Bogaerts [50]	High symptom reporters	Rebreathing task	58	-.0368
Bräscher [80]	Healthy participants with varying levels of somatic symptoms	Heartbeat mental tracking task	60	-.0450
Ferentzi [81]	Healthy participants with varying levels of somatic symptoms	Heartbeat mental tracking task; elbow joint position matching task	109	.0115
Haenen [82]	Hypochondriasis	Tactile two-point discrimination task	51	.1043
Katzer [29]	Healthy participants with varying levels of somatic symptoms/illness anxiety	SSDT (tactile threshold)	67	.1554
Katzer [19]	Somatoform disorders	SSDT (tactile threshold)	65	.2605
Krautwurst [58]	Healthy participants varying levels of somatic symptoms/illness anxiety	Signal detection task for NSCF; heartbeat mental tracking task	100	-.0909
Krautwurst [83]	Illness anxiety disorder	Signal detection task for NSCF; heartbeat mental tracking task	107	.1627
Lee [84]	Somatic symptom disorder	Heartbeat perception task	43	.0087
Meyerholz [85]	Healthy participants with varying levels of somatic symptoms	Heartbeat mental tracking task	100	.0100
Miles [86]	High symptom reporters	Rubber hand illusion task	40	.2774
Perepelkina [87]	Somatoform disorder	Rubber and virtual hand illusion task	33	.0055
Petersen [18]	High symptom reporters	Breathing resistance task	50	.2616
Pollatos [25]	Multisomatoform disorder	Heartbeat mental tracking task	46	-.3008
Rodic [88]	Healthy participants with varying levels of somatic symptoms	Tactile perception task	179	-.0804
Sachse [28]	Psychological Factors Affecting Other Medical Conditions	Heartbeat tracking task, heartbeat mental tracking task	66	-.3377
Sarnoch [89]	High symptom reporters	Muscle tension perception task	13	-.5342
Schäfer [23]	Somatization disorder, pain disorder, undifferentiated somatoform disorder	Heartbeat mental tracking task	50	-.0121
Scholz [20]	High symptom reporters	Muscle tension perception task	40	.2939
Schonecke [27]	Functional cardiac disorder	Heartbeat discrimination task	49	-.5159
Schröder [24]	Noncardiac chest pain		91	-.0235
Schulz [90]	High symptom reporters	Heartbeat mental tracking task; heartbeat discrimination task	58	-.0075
Weiss [26]	Multisomatoform disorder	Heartbeat mental tracking task	60	-.3439
Witthöft [91]	Healthy participants with varying levels of somatic symptoms	Heartbeat mental tracking task	316	-.0300
	Functional syndromes			
Akyol [92]	Fibromyalgia	Knee repositioning task	105	-.0296
Anastasopoulos [93]	Spasmodic torticollis	Subjective vertical task	51	-.1681
Bara-Jimenez [94]	Focal dystonia of the right hand	Tactile temporal discrimination task	27	-.4369
Bara-Jimenez [95]	Focal dystonia of the right hand	Spatial localization task, tactile gap detection task	30	-.5038
Bardal [51]	Fibromyalgia	Shoulder repositioning task	50	-.0740
Borg [96]	Fibromyalgia	Heartbeat mental tracking task	42	.1260
Brun [97]	Fibromyalgia	Arm positioning matching task	40	.0387
Celenay [98]	Fibromyalgia	Trunk repositioning task	30	-.5260
Cheng [99]	Neck pain	Head repositioning task	24	-.4071
Demartini [100]	Psychogenic non-epileptic seizures and functional motor symptoms	Heartbeat mental tracking task	40	-.0939
Demartini [101]	Functional motor symptoms	Heartbeat mental tracking task	40	-.0892
De Pauw [102]	Cervical dystonia	Head repositioning task	94	-.2635
De Zoete [103]	Neck pain	Head and trunk repositioning task	100	-.0482
Dumas [104]	Cervicogenic headache, migraine	Head repositioning task	37	-.0890
Duschek [105]	Fibromyalgia	Heartbeat mental tracking task	76	-.2498
Edmondston [106]	Postural neck pain	Head, neck and shoulder repositioning task	43	-.0569
Elsig [107]	Neck pain	Tactile discimination task; head repositioning task	60	-.2509
Fiorio [108]	DYT1 manifesting dystonia	Tactile temporal discrimination task	39	.1321
Fiorio [109]	Blepharospasm	Tactile temporal discrimination task	20	-.7360
Fiorio [110]	Focal hand dystonia, non-hand dystonia	Rubber hand illusion task	22	-.5349
Gajdos [111]	High gastrointestinal symptom reporters	Heartbeat mental tracking task	72	.1170
Goncalves [112]	Chronic neck pain	Head repositioning task	66	-.2657
Grip [113]	Neck pain	Head repositioning task	43.75^a^	-.0667
Jungilligens [114]	Dissociative seizures	Heartbeat mental tracking task	40	-.0931
Katschnig [115]	Fixed Dystonia	Tactile temporal discrimination task	21	-.4058
Koreki [116]	Functional seizures	Heartbeat mental tracking task; heartbeat discrimination task	71	-.2657
Kristjansson [117]	Neck pain	Head repositioning task; trunk rotation task; figure-of-eight movement task	41	-.1103
Lee [118]	Non-clinical daily and weekly neck pain	Head repositioning task	85.5^a^	-.2594
Marinelli [119]	Cervical Dystonia	Reaching movement task	20	-.5466
Morgante [120]	Primary torsion dystonia and psychogenic dystonia	Tactile temporal discrimination task	26	-.9622
Nijs [121]	Chronic fatigue syndrome	Leg repositioning task	137	-.1475
Pick [122]	Functional neurological disorder	Heartbeat mental tracking task	39	.0090
Pinsault [123]	Neck pain	Head repositioning task	14	-.4602
Ricciardi [124]	Functional movement disorders	Heartbeat mental tracking task	33	-.8568
Rost [125]	Fibromyalgia	Heartbeat mental tracking task	92	.1413
Sanger [126]	Focal hand dystonia	Tactile temporal discrimination task	19	-.5651
Scontrini [127]	Blepharospasm, cervical, hand, and laryngeal dystonia	Tactile temporal discrimination task	41	-.5001
Sjölander [128]	Neck pain	Head repositioning task	25	-.2428
Tinazzi [129]	Generalized dystonia, hand dystonia, and segmental dystonia involving the right arm and trunk	Tactile temporal discrimination task	16	-.7420
Tinazzi [130]	Generalized dystonia, hand dystonia, and segmental dystonia involving the right arm and trunk	Tactile temporal discrimination task	22	-.8582
Ulus [131]	Fibromyalgia	Knee repositioning task	60	-.0541
Valenzuela-Moguillansky [132]	Fibromyalgia	Heartbeat detection task	59	-.0174
Woodhouse [133]	Chronic neck pain	Head repositioning task	114	-.1335

*Note*. ^a^ Sample sizes averaged over tasks/conditions.

Moderator analyses showed that sample (clinical or non-clinical) was a significant moderator, *Q* = 5.19, *p* = .023, *I*^*2*^ = 84.44%, *H*^*2*^ = 6.43. Likewise, diagnosis (functional syndromes, somatic symptom disorder, illness anxiety disorder) had a significant impact on the population effect size, *Q* = 8.94, *p* = .003, *I*^*2*^ = 83.68%, *H*^*2*^ = 6.13. The effect size was not significantly affected by interoceptive domain (visceral/muscle perception, tactile, proprioceptive), *Q* = 0.46, *p* = 0.500, *I*^*2*^ = 85.49%, *H*^*2*^ = 6.89, or task type (signal detection, thresholding procedure, heartbeat mental tracking, position sense, rubber hand illusion, and correlational tasks), *Q* = 0.00, *p* = 0.947, *I*^*2*^ = 84.92%, *H*^*2*^ = 6.63.

Subgroup analyses were conducted for all levels of the significant moderators sample and diagnosis (see Table 2). Effect size for IAcc was considerably higher in clinical samples (*r*_*z*_ = -.251) than non-clinical samples (*r*_*z*_ = -.014), with a significantly associated *z-*score but also high heterogeneity in studies with clinical samples. Regarding diagnosis, only studies with functional syndrome samples showed a significant effect size of *r*_*z*_ = -.308.

**Table 2:** Results of separate random-effects subgroup analyses for interoceptive accuracy according to diagnosis, task, and sample.

Subgroup	*k*	95% confidence interval for estimated *r*_*z*_			*z*	*τ* ^*2*^	*Q* ^a^	*I* ^*2*^	*H* ^*2*^
		lower	mean	upper					
Sample									
Clinical	54	-.355	-.251	-.146	-4.71***	.126	273.85***	85.91	7.10
Non-clinical	14	-.077	-.014	.050	-0.42	.002	20.38	16.43	1.20
Diagnosis									
SSD	22	-.137	-.049	.038	-1.11	.026	53.20***	65.15	2.87
IAD	6	-.037	.053	.142	1.15	.002	5.76	16.55	1.20
FS	43	-.430	-.308	-.186	-4.96***	.137	216.84***	86.30	7.30

*Note*. SSD = somatic symptom disorder; IAD = illness anxiety disorder, FS = functional syndrome

^a^ *df* = *k–* 1

*p < .05

**p < .01

***p < .001

### Effects of RB

Effect sizes are shown in Table 3. For measures of RB, there was an estimated mean *r*_*z*_ of -.163, with lower and upper confidence bounds of -.252 and -.075, respectively. The associated *z*-score was significant, *z* = -3.63, *p* < .001. The estimated between study variance τ^*2*^ was .002, *I*^*2*^ = 14.64%, *H*^*2*^ = 1.17, and *Q* statistics of homogeneity were not significant, *Q* = 7.62, *df* = 7, *p* = .367).

**Table 3:** Effect sizes of response bias (unstandardized *r*) for included studies.

First author (reference)	Diagnosis/symptoms of experimental group	Task	*N*	RB
Brown [30]	High symptom reporters	SSDT	80	-.2022
Katzer [29]	Healthy participants with varying levels of somatic symptoms/illness anxiety	SSDT	67	-.3550
Katzer [19]	Somatoform disorders	SSDT	65	-.1264
Krautwurst [58]	Healthy participants with varying levels of somatic symptoms/illness anxiety	Signal detection task for NSCF	100	-.1104
Krautwurst [83]	Illness anxiety disorder	Signal detection task for NSCF	107	-.2505
Petersen [18]	High symptom reporters	Breathing resistance task	50	-.1597
Schäfer [23]	Somatization disorder, pain disorder, undifferentiated somatoform disorder	Heartbeat discrimination task	50	-.1285
Schröder [24]	Noncardiac chest pain	Heartbeat discrimination task	91	.0352

*Note*. RB = response bias

#### Risk of bias evaluation

The risk of bias of studies included into this systematic review was quite heterogeneous (see S4 Table for details of the risk of bias assessment). Across all studies and criteria, 51.4% were rated “yes” (indicating low risk of bias). Risk of bias indicators differed across criteria: For example, 84.1% described or cited a protocol for IAcc assessment in their study, but less than half (46.4%) provided statistics for age matching their study groups. Risk of bias indicators also differed with regard to study sample: For example, more studies examining somatic symptom disorder and illness anxiety disorder samples described replicable sampling methods than studies examining functional syndrome samples (62.7% vs. 38.1%).

#### Publication bias analyses

**Fig 2:**
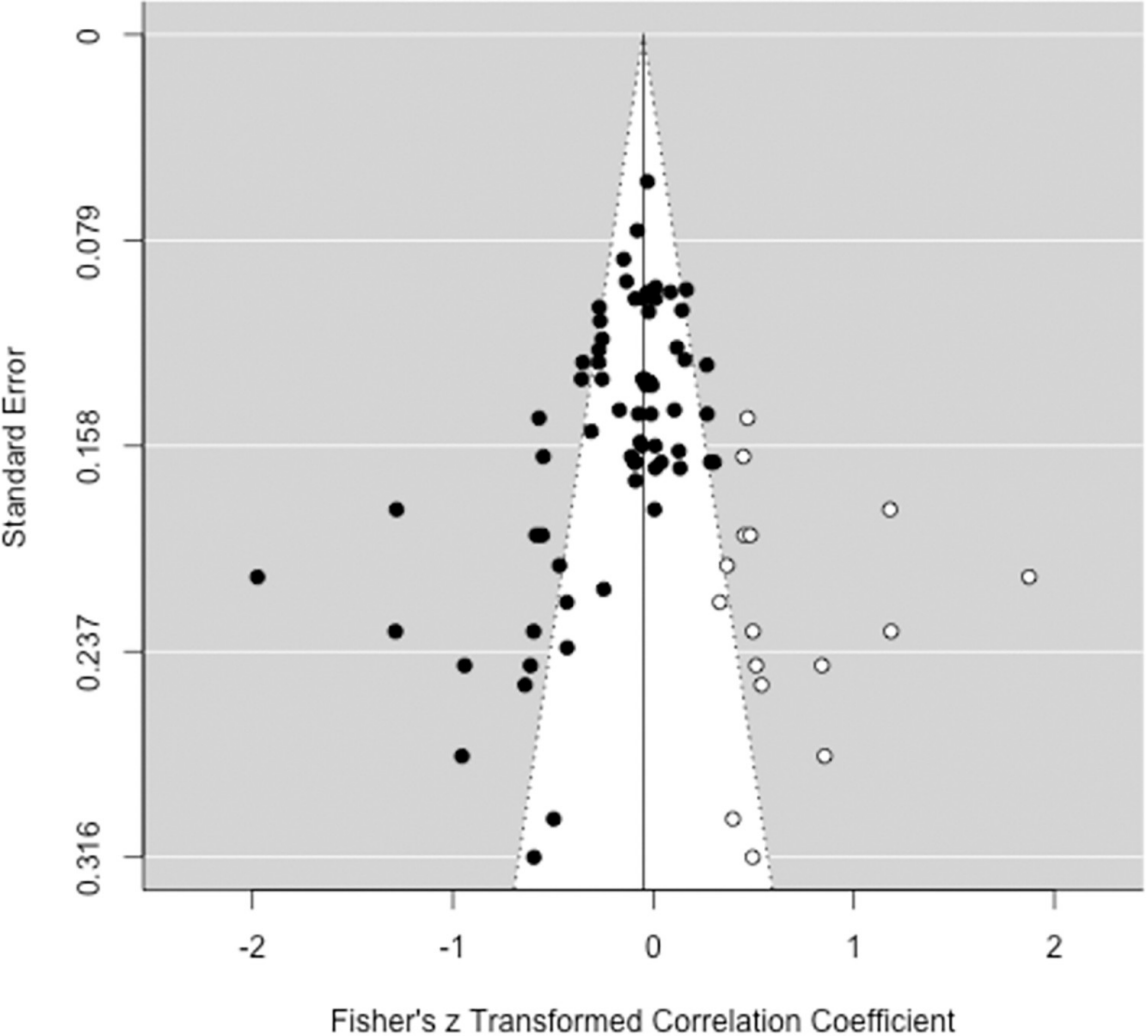
Funnel plot with trim and fill method applied to *z*-transformed correlations and standard errors of interoceptive accuracy (*k* = 68). Created with the metafor package [54] for R (Version 3.3.1).

Regarding IAcc outcomes, there was noticeable asymmetry in the funnel plot of studies’ *z*-standardized effect estimates and standard errors (see Fig 2). By means of the trim and fill method [68], 16 data points were augmented on the right side of the funnel plot, which shows that studies with negative correlations, some of them very strong, are overrepresented. It should however be noted that the trim and fill method is problematic when moderator effects are expected [54]. Between-study heterogeneity might have had an effect on the distribution of data. In our selection, four studies that reported effect sizes < -.5 were conducted by the same workgroup [109, 120, 129, 130] with similar patient samples (dystonia). Similarly, with few exceptions, the positive correlations are based on samples with somatic symptom disorder or illness anxiety disorder, suggesting that subgroup effects skew the plot. Egger’s regression test confirmed significant funnel plot asymmetry, *z* = -5.444, *p* < .001.

Rosenthal’s [70] fail safe *N* test revealed that 2232 new, unpublished, filed, or unretrieved studies would be required to turn the significant result into a non-significant one. Begg and Mazumdar rank correlation test for a random-effects model showed significant publication bias across all studies (*τ*_*B*_ = -.330, *p* < .001). Sensitivity analysis based on Vevea and Woods [72] revealed minor changes in population effect size estimates (unadjusted *r*_*z*_ = -.195) in case of moderate and severe two-tailed selection (*r*_*z*_ = -.181; *r*_*z*_ = -.165), but considerable changes in case of moderate and severe one-tailed selection (*r*_*z*_ = -.271; *r*_*z*_ = -.704).

**Fig 3:**
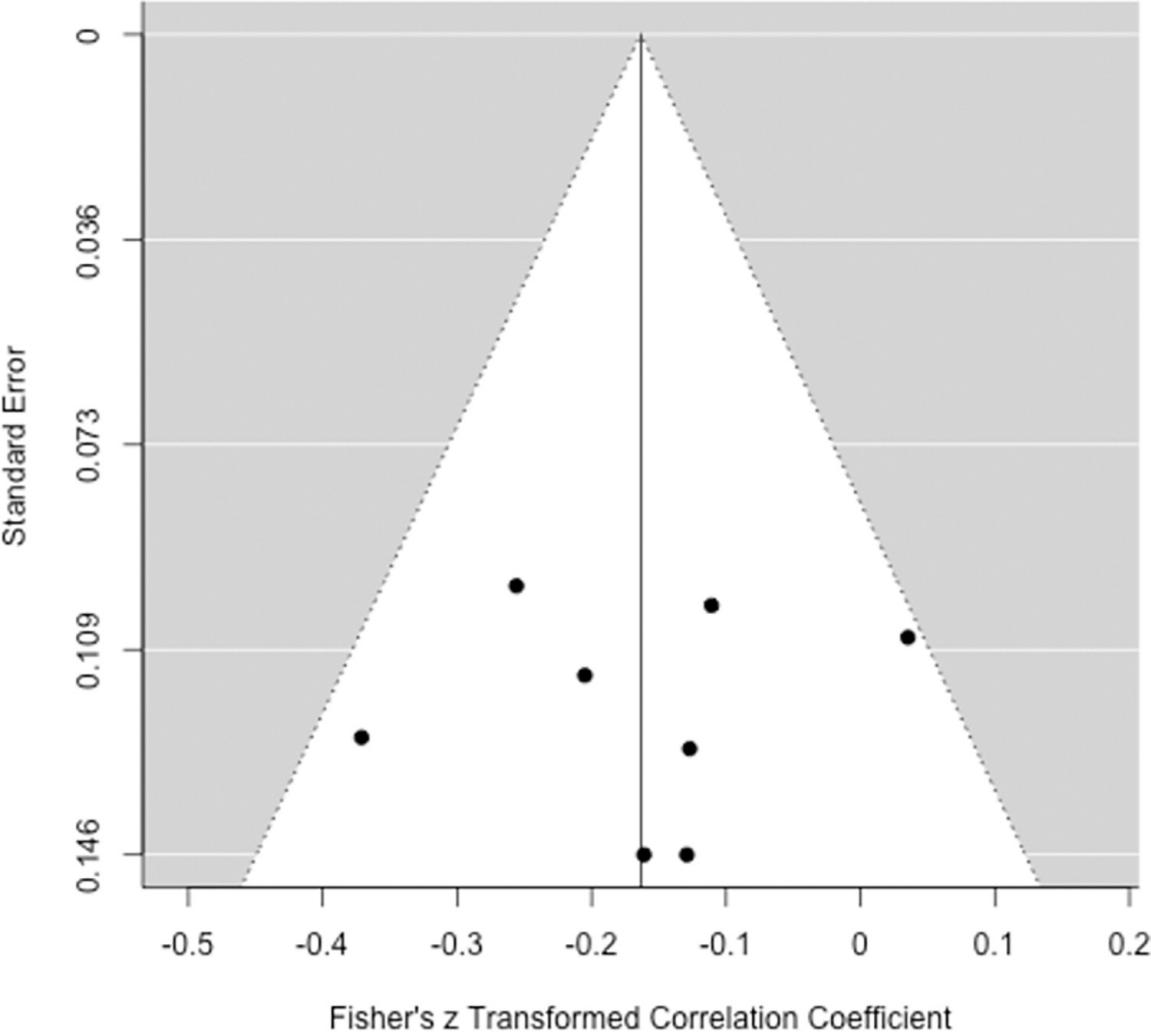
Funnel plot applied to *z*-transformed correlations and standard errors of response bias (*k* = 8). No data points were augmented by the trim and method. Created with the metafor package [54] for R (Version 3.3.1).

For RB outcomes, the funnel plot with *z* transformed correlations was symmetric and no data points were augmented using the trim and fill method (see Fig 3). Neither Egger’s regression test, *z* = -0.19, *p* = .842, nor Begg and Mazumdar rank correlation test, *τ*_*B*_ = -.036, *p* = .901, showed evidence of publication bias. Rosenthal’s [70] fail safe *N* was 38. Sensitivity analysis showed the largest changes in effect size estimation for severe one-tailed publication bias (*r*_*z*_ = —.236, unadjusted *r*_*z*_ = -.163) and smaller changes for moderate one-tailed (*r*_*z*_ = -.173) as well as moderate and severe two-tailed selection (*r*_*z*_ = -.148, *r*_*z*_ = -.124).

## Discussion

### Interoceptive accuracy and response bias

We found a small overall effect size of reduced IAcc, which was moderated by diagnosis and by whether the sample was clinical or non-clinical. Subgroup analyses showed that while IAcc was significantly reduced in functional syndromes, it was not altered in somatic symptom disorder and illness anxiety disorder. This contradicts models that assume lower perceptual thresholds for body signals in these disorders. In line with this, studies homogeneously showed a more liberal RB, reflecting a “better safe than sorry” approach [18]. A liberalization of decision strategies in the formation of somatic symptom and related disorders is compatible with predictive coding theory: symptom report decoupled from sensory input should occur when learned knowledge about the world (“priors”) predicts the presence of symptoms/stimuli with a high level of confidence [16]. Over the course of time, the experience of symptoms may increasingly depend on contextual cues that confirm the underlying disease model rather than on perceived physiological sensations [134–142]. Assuming that a sensation is a “symptom” rather than a “benign sensation” can then turn into the prior with the highest precision in somatic symptom and related disorders.

The overall effect of reduced IAcc was characterized by high heterogeneity, which was only partly resolved by including moderators. Therefore, the general and subgroup effects of IAcc have to be interpreted with caution. Possible explanations for the heterogeneity will be discussed in the following.

#### Sources of heterogeneity in interoceptive accuracy: Differences between diagnostic groups

There was a medium effect size of reduced IAcc for functional syndromes, but effect sizes around zero for somatic symptom disorder or illness anxiety disorder samples. Differences in IAcc between diagnoses are surprising, considering substantial overlap of disorders that are not attributable to verified organic dysfunctions [41].

These differences could be due to underlying pathological differences between diagnostic groups. IAcc measurements might be affected by specific symptoms. In functional syndromes, the symptoms often affect one or few body parts and remain relatively stable across time. In somatic symptom and related disorders, symptoms are highly variable and typically co-occur [143]. In fact, DSM-IV diagnosis of somatoform disorder required the co-occurrence of symptoms in at least four different body sites or functions [75]. Yet, more importantly, illness anxiety does not necessarily involve any somatic symptoms. Therefore, abnormalities of IAcc in somatic symptom and related disorders would have to be very generalized in order to produce a common effect. In contrast, specific distortions in functional syndromes such as neck pain might more easily result in measurably lower IAcc assessed in this domain. It should also be noted that dystonia was by far the most frequently diagnosed disorder and might have had a particularly strong impact on the mean effects size in functional syndromes.

We believe that interoceptive domain and task type must be considered in the interpretation of our results despite nonsignificant moderator effects, because there was a strong entanglement of the three moderators diagnosis, task type, and interoceptive domain. For example, studies investigating somatic symptom disorder and illness anxiety disorder predominantly used visceral tasks, while none of the studies investigating dystonia did so. Studies assessing functional syndromes often measured IAcc in affected body parts, such as using head repositioning tasks in neck pain. In this case, muscle tension in case of neck pain might impair patients’ abilities to perceive interoceptive signals in the neck region. Arguably, individual symptoms and IAcc measurements are therefore more closely related in studies assessing functional syndromes, and impaired IAcc might result from physiological dysfunction rather than generally impaired perception of body signals. In contrast, IAcc outcomes for body regions specifically affected by symptoms of somatic symptom disorder were relatively rare [with few exceptions: e.g. cardiac vs. non-cardiac chest pain, 24, or heartbeat detection tasks in functional cardiac disorder, 27]. On the other hand, only about half of the dystonia studies assessed IAcc in the affected body part and one study provided direct evidence for a generalized proprioceptive impairment by comparing affected and non-affected body parts [99]. Interestingly, most theories conceptualize interoception as a homogenous construct across assessment domains [144]. However, while a few studies found cross-modal intercorrelations [gastrointestinal and cardiac domain: 145, 146, cardiac domain and body ownership: 147, cardiac and respiratory domain: 148], most others did not [skin conductance and cardiac domain: 58, 83, 149, 150, cardiac, sweat gland, and respiratory perception: 151, propriocption as well as cardiac, gastric, and taste perception: 152]. This could also be due to the fact that there are hardly any studies that measure body domains with comparable task types (type of demand, difficulty).

There was a similar overlap between task type and diagnosis. For example. with one exception, position sense tasks were only used in functional syndrome samples. Signal detection tasks were used in all diagnostic groups, but with different paradigms (e.g., tactile spatial localization tasks and temporal discrimination tasks were only assessed in functional syndrome samples). Few studies had used correlational tasks or the rubber hand illusion task. On the other hand, the mental tracking task was used across diagnoses.

#### A short evaluation of conducted task types

It is important to acknowledge that all task types have different advantages and disadvantages. Test and retest reliability of position sense tasks was merely moderate [153] and results depended on outcome variable [154]. Short term reliability of tactile signal detection tasks and thresholding procedures was good [19, 29], but might vary depending on examined parts of the skin or when assessed long term [155]. An external stimulation is necessary in theses task types, which excludes them from narrow definitions of interoception.

Although the separation of sensitivity and bias according to signal detection approaches is of especial interest for testing etiological assumptions for somatic symptom and related disorders, most task types do not allow this differentiation. For example, the mental tracking task is reliable and easy to administer, but the heartbeat perception score is an amalgam of sensitivity and response bias, with liberal decisions resulting in increased accuracy scores [156]. A lack of group differences in this task may be because poorer sensitivity is compensated for by increased liberality. The validity of this task type has consequently been questioned [157, 158]. Discrimination tasks, on the other hand, have been criticized for their high level of difficulty, leading to low IAcc scores with low variability [18, 157]. In a similar vein, the signal detection task in the electrodermal body domain is only well suited for specific samples due to its high degree of difficulty and temporal length [58, 83, 159, 160].

### Strengths and limitations of this systematic review

Taken together, the results summarized in our review do not confirm assumptions about abnormalities regarding IAcc for somatic symptom disorder or illness anxiety disorder. We did, however, find abnormalities of IAcc for a broad range of functional syndromes, implying IAcc to be a more relevant underlying mechanism in these disorders. Due to high heterogeneity these results have to be interpreted cautiously. To our knowledge, this is the first systematic review that systematically covers a broad variety of IAcc concepts in various functional and somatic symptoms and disorders.

When evaluating our results, both risks and advantages of bringing together heterogeneous samples and methods in meta-analyses should be carefully considered [55]. In our selection of studies, participants differed broadly in diagnoses. Methods of assessing IAcc differed considerably between diagnostic groups. Some of them, such as heartbeat detection tasks, have been criticized because of their psychometric limitations [161]. We dealt with this issue by considering moderators and analyzing subsamples. However, we cannot rule out that these factors affected our results.

We only included “neutral” task conditions into the systematic review. However, interoceptive measures might be more clinically sensitive if related to perturbations of physiological functioning [compare 9]. For example, healthy individuals with high negative affectivity were significantly less accurate in estimating breathing sensations presented in a distressing frame than a pleasant frame, while framing did not have an impact on accuracy in individuals with low negative affectivity [135]. It is conceivable that IAcc increases or decreases in a threatening context such as pain induction because of its inherent association with negative affectivity [46]. Previous research indicated lower pain thresholds in patients with somatoform pain disorders [162] and illness anxiety disorder [163]. Then again, no abnormalities of pain thresholds were found in patients with multisomatoform disorder [164] and somatoform disorder [26] in comparison to healthy controls. Evidence is yet too sparse to confirm differences of IAcc between “negative affect” and “neutral” conditions.

Another possible limitation relates to risk of bias in the included studies. All in all, methodological quality was reasonably good for the majority of studies. However, we did find risk of bias for some aspects that would be easy to prevent (such as describing replicable sampling methods).

An issue relevant to all meta-analyses is publication bias. Despite a comprehensive literature search considering grey literature such as dissertations, we found evidence of publication bias in our sample, with an overrepresentation of strong negative effect sizes. Therefore, we cannot rule out that the true effect of IAcc is lower than calculated in this meta-analysis.

Finally, our study sample regarding RB in somatic symptom and related disorders is small. Our findings with regard to a more liberal RB are therefore preliminary, and more data-driven studies are needed to extend these findings.

### Future directions

This systematic review focused on two facets of interoception: IAcc and RB. While our findings contradict the assumption of generally altered IAcc, somatic symptom and related disorders patients might show higher or lower IAcc under certain conditions (e.g., when symptom schemata are activated), or in certain domains (e.g., those affected by symptoms). Besides, IAcc and RB are not mutually exclusive and have to be addressed independently. They might interact differentially depending on psychological factors as affect or cognition [18]. For example, a positive relation of IAcc and RB was shown in case of increased uncertainty about stimuli [18]. For such highly ambiguous stimuli, extreme decision strategies can be successful and adaptive [18].

There is a clear need for high-quality multilevel studies [9] to tackle different interoceptive domains and link various aspects of interoception. Interoception is a multifaceted process ranging from peripheral signal perception to higher cognitive processes such as attention, attribution, and decision-making [9]. Ideally, future research will be able to integrate findings on lower level processing [165] and higher-level processing [e.g. the impact of categorization on interoceptive processing, 136]. Recent advances in interoceptive techniques using sensing perturbation [166, 167] could provide further insight into bottom-up interoceptive processes, while manipulations of expectations [e.g. 50, 135] could help to explore top-down interoceptive processing in somatic symptom and related disorders. The predominance of interoceptive measures in certain body domains within diagnostic categories (e.g., proprioceptive tasks in functional syndromes) should be addressed by using a greater variety of tasks in different interoceptive domains. Furthermore, a development of signal detection tasks with comparable difficulty for different body domains would be desirable (for a new approach in the cardiovascular body domain see [156]). The risk of bias criteria assessment can inform the experimental set ups of future studies. However, even within task types, different forms of implementation can lead to strongly diverging results [168]. Therefore, achieving a better comparability of different studies would also require the research field to agree on relevant control variables and quality standards [e.g., 168].

Furthermore, future research should follow up on first findings of beneficial effects of biofeedback training [21, 85] and interoceptive exposure [169] in patients with somatic symptom and related disorders. Importantly, researchers should refer to the proposed taxonomies of interoception [9] to allow an integration of findings on the various dimensions of interoceptive processing.

## Conclusions

In this systematic review and meta-analysis, IAcc was assessed in somatic symptom and related disorders in the light of different theoretical approaches. We found diminished IAcc in functional syndromes and a more liberal RB in somatic symptom disorders and illness anxiety disorder. These findings are consistent with the predictive coding theory, which highlights a decoupling of somatosensory input and the perception of body sensations. However, the RB effect was only based on the few studies that had distinguished between IAcc and RB in their experimental setup. Consequently, future research should consider this distinction to further elucidate the relationship between these two facets of interoception.

Finally, we would like to encourage researchers to use different tasks and assess various interoceptive domains. Future study designs should address interoception both in contexts closely and more distantly related to potential dysfunctions. Using multifaceted approaches will help to provide ecologically valid results and to explore the relevance of symptom specificity as originally suggested by Malmo and Shagass [170].

## Supporting information

S1 File PRISMA 2009 checklist. (DOCX)

S1 Table Specification of literature search. TS = topic, TI = title.
(DOCX)

S2 Table Study characteristics and results. CFS = chronic fatigue syndrome, CFS-APQ = Chronic Fatigue Syndrome Activities and Participation Questionnaire, CG = control group, CSD = Checklist for Symptoms in Daily Life, EG = experimental group, FIQ = Fibromyalgia Impact Questionnaire, HAF-17 = Cardiac Anxiety Questionnaire [Herzangstfragebogen], IAcc = interoceptive accuracy, IQR = interquartile range, M = mean, Mdn = Median, MIHT = Multidimensional Inventory of Hypochondriacal Traits, NA = not available, n.s. = not significant, NSCF = nonspecific skin conductance fluctuations, PHQ-15/PHQ-D = Patient Health Questionnaire, RB = response bias, SD = standard deviation, SDQ/SDQ-20 = Somatoform Dissociation Questionnaire, SE = standard error, SOMS = Screening for Somatoform Symptoms, SSAS = Somatosensory Amplification Scale, SSDT = Somatic Signal Detection Task, TWSTRS = Toronto Western Spasmodic Torticollis Rating Scale, WGO-IBS = World Gastroenterology Organisation–Irritable Bowel Syndrome Questionnaire, WI = Whiteley Index. ^a^ Number of participants included into data analysis. ^b^ These numbers were calculated on the basis of data supplied by the authors. ^c^ Only tasks/outcomes included in effect size calculation are listed here. ^d^ Number of outcomes included into data analysis. ^e^ < indicates a more liberal response bias, > a more conservative response bias in the EG compared to the CG. ^e^ Refers to the total sample. ^f^ Difference between target and actual position.
(DOCX)

S3 Table Descriptions of tasks. (DOCX)

S4 Table Risk of bias assessment. Yes (low risk of bias); no (high risk of bias);? (unclear); NA (not applicable).
(DOCX)
