# Supplementary material for: Interoceptive accuracy and bias in somatic symptom disorder, illness anxiety disorder, and functional syndromes: A systematic review and meta-analysis
Source: PLoS One. 2022 Aug 18;17(8):e0271717. doi: 10.1371/journal.pone.0271717 (PMC9387777; doi:10.1371/journal.pone.0271717)
Supplement: S1 Table — TS = topic, TI = title. (DOCX) [file pone.0271717.s002.docx]

**S1 Table**

| Database | Search terms | Conjunction of search terms |
| --- | --- | --- |
| Medline | 1. (interocept* OR propriocept*)  2. (somatization OR somatisation OR “medically unexplained“ OR “bodily distress“ OR somatoform OR psychosomatic OR hypochondr* OR “health anxiety“ OR "functional disorder" OR "functional syndrome" OR "functional symptom" OR idiopathic OR psychogenic OR nonorganic OR non-organic OR fibromyalgia OR "bowel disease" OR "bowel syndrome" OR "chronic fatigue" OR headache OR "functional constipation" OR "functional dyspepsia" OR "functional dyspnea" OR "functional chest pain" OR "functional limb weakness" OR "functional tremor" OR "functional dystonia" OR "functional walking problems" OR "functional neurological disorder" OR "functional movement disorder" OR conversion)  3. (review OR meta analysis OR meta-analysis) | 1. (Title/Abstract) AND 2 (Title/Abstract) NOT 3 (Title) |
| ProQuest | interoception OR proprioception OR interoceptive OR proprioceptive AND (somatization OR somatisation OR medically unexplained OR bodily distress OR somatoform OR psychosomatic OR hypochondriasis OR health anxiety OR functional disorder OR functional syndrome OR functional symptom OR idiopathic OR psychogenic OR nonorganic OR non-organic OR fibromyalgia OR bowel disease OR bowel syndrome OR chronic fatigue OR headache OR functional constipation OR functional dyspepsia OR functional dyspnea OR functional chest pain OR functional limb weakness OR functional tremor OR functional dystonia OR functional walking problems OR functional neurological disorder OR functional movement disorder OR conversion) |  |
| PsycINFO | 1. (interocept* OR propriocept*)  2. (somatization OR somatisation OR „medically unexplained“ OR „bodily distress“ OR somatoform OR psychosomatic OR hypochondr* OR „health anxiety“ OR "functional disorder" OR "functional syndrome" OR "functional symptom" OR idiopathic OR psychogenic OR nonorganic OR non-organic OR fibromyalgia OR "bowel disease" OR "bowel syndrome" OR "chronic fatigue" OR headache OR "functional constipation" OR "functional dyspepsia" OR "functional dyspnea" OR "functional chest pain" OR "functional limb weakness" OR "functional tremor" OR "functional dystonia" OR "functional walking problems" OR "functional neurological disorder" OR "functional movement disorder" OR conversion)  3. (review OR meta analysis OR meta-analysis) | 1 (Abstract) AND  2 (Abstract) NOT  3 (Title) |
| Web of Science | TS=(interocept* OR propriocept*) AND TS=(somatization OR somatisation OR "medically unexplained" OR "bodily distress" OR somatoform OR psychosomatic OR hypochondr* OR „health anxiety“ OR idiopathic OR psychogenic OR nonorganic OR non-organic OR fibromyalgia OR "bowel disease" OR "bowel syndrome" OR "chronic fatigue" OR headache OR "functional constipation" OR "functional dyspepsia" OR "functional dyspnea" OR "functional chest pain" OR "functional limb weakness" OR "functional tremor" OR "functional dystonia" OR "functional walking problems" OR "functional neurological disorder" OR "functional movement disorder" OR conversion) NOT TI=(review OR meta analysis OR meta-analysis) |  |
