# Supplementary material for: Interoceptive accuracy and bias in somatic symptom disorder, illness anxiety disorder, and functional syndromes: A systematic review and meta-analysis
Source: PLoS One. 2022 Aug 18;17(8):e0271717. doi: 10.1371/journal.pone.0271717 (PMC9387777; doi:10.1371/journal.pone.0271717)
Supplement: S2 Table — CFS = chronic fatigue syndrome, CFS-APQ = Chronic Fatigue Syndrome Activities and Participation Questionnaire, CG = control group, CSD = Checklist for Symptoms in Daily Life, EG = experimental group, FIQ = Fibromyalgia Impact Questionnaire, HAF-17 = Cardiac Anxiety Questionnaire [Herzangstfragebogen], IAcc = interoceptive accuracy, IQR = interquartile range, M = mean, Mdn = Median, MIHT = Multidimensional Inventory of Hypochondriacal Traits, NA = not available, n.s. = not significant, NSCF = nonspecific skin conductance fluctuations, PHQ-15/PHQ-D = Patient Health Questionnaire, RB = response bias, SD = standard deviation, SDQ/SDQ-20 = Somatoform Dissociation Questionnaire, SE = standard error, SOMS = Screening for Somatoform Symptoms, SSAS = Somatosensory Amplification Scale, SSDT = Somatic Signal Detection Task, TWSTRS = Toronto Western Spasmodic Torticollis Rating Scale, WGO-IBS = World Gastroenterology Organisation–Irritable Bowel Syndrome Questionnaire, WI = Whiteley Index. a Number of participants included into data analysis. b These numbers were calculated on the basis of data supplied by the authors. c Only tasks/outcomes included in effect size calculation are listed here. d Number of outcomes included into data analysis. e < indicates a more liberal response bias, > a more conservative response bias in the EG compared to the CG. e Refers to the total sample. f Difference between target and actual position. (DOCX) [file pone.0271717.s003.docx]

**S2 Table**

| Authors (Year) | Sample size^a^ | Age^b^ | % of  females included^b^ | EG diagnosis/ symptoms | Task^c^ | Outcome measure | N^d^ | IAcc result | RB result^e^ | | | Secondary Outcomes |
| --- | --- | --- | --- | --- | --- | --- | --- | --- | --- | --- | --- | --- |
| Somatic symptom disorder / illness anxiety disorder | | | | | | | | | | | | |
| Barsky, Brener, Coeytaux, and Cleary (1995) | EG: 55  CG: 50 | EG: *M* = 46.6 (*SD* = 15.4)  CG: *M* = 55.0 (*SD* = 15.5) | EG: 43.0  CG: 39.0 | Hypochondriasis (DSM-III-R) | Heartbeat discrimination task (1) | Interquartile ranges | 1 | EG = CG |  | | | EG: positive correlation for IAcc x WI |
| Bogaerts et al. (2008) | EG: 34  CG: 40 | Range 18-26 | 100.0 | High vs. low symptom reporters (CSD) | Rebreathing task | Correlations between subjective rating and minute ventilation in neutral frame trials | 2 | EG = CG |  | | |  |
| Bräscher, Schulz, Van den Bergh, and Witthöft (2020) | 60 | *M* = 27.3 (*SD* =7.38) | 63.0 | Healthy participants (CSD) | Heartbeat mental tracking task (2) | Relative difference between actual and counted number of heartbeats | 1 | No significant correlation of heartbeat perception score x CSD |  | | |  |
| Brown, Brunt, Poliakoff, and Lloyd (2010) | EG: 40  CG: 40 | *M* = 20.61 (*SD* = 3.96) | 61.0 | High vs. low symptom reporters (SDQ-20) | SSDT | *c* | 1 |  | EG < CG | | |  |
| Ferentzi et al. (2018) | 105 | *M* = 21.9 (*SD* =3.6) | 46.5 based on (*N* = 124) | Healthy participants (PHQ-15) | Heartbeat mental tracking (2) | Relative difference between actual and counted number of heartbeats | 2 | EG = CG |  | | |  |
|  | 113 | *M* = 21.9 (*SD* = 3.6) | 46.5 based on (*N* = 124) | Healthy participants (PHQ-15) | Elbow joint position matching task | Proprioceptive error |  | EG= CG |  | | |  |
| Haenen, Schmidt, Schoenmakers, and van den Hout (1997) | EG: 25  CG: 23 | EG: *M* = 49.0 (*SD* = 12.1); CG: *M* = 49 (*SD* = 12.1) | EG: 55.6  CG: 55.6 | Hypochondriasis (DSM-III-R) | Two-point discrimination task | Tactile threshold | 1 | EG = CG |  | | |  |
| Katzer, Oberfeld, Hiller, and Witthöft (2011)Katzer, Oberfeld (3) | 67 | *M* = 23.2 (*SD* = 4.8) | 79.1 | Healthy participants (PHQ-15, WI) | SSDT | Pre-SSDT tactile detection threshold; *c* | 2 | No significant correlations of tactile thresholds x PHQ-15 and WI | Significant negative correlations of *c* x PHQ-15 and WI | | |  |
| Katzer, Oberfeld, Hiller, Gerlach, and Witthöft (2012) | EG: 33  CG: 32 | EG: *M* = 43.4 (*SD* = 9.9)  CG: *M* = 41.7 (*SD* = 11.5) | EG: 72.7  CG: 68.8 | Somatoform disorders (DSM-IV) | SSDT | Pre-SSDT tactile detection threshold; *c* | 4 | EG < CG | EG = CG | | | EG: Significant positive correlation of IAcc x PHQ-15 and WI  CG: Significant positive correlations of RB in 1^st^ test half x SOMS gastrointestinal scale; RB in 2^nd^ test half x SOMS pain scale and pseudoneurological scale; IAcc x SOMS pseudoneurological scale |
| Krautwurst, Gerlach, Gomille, Hiller, and Witthöft (2014) | 100 | *M* = 23.7 (*SD* = 3.4) | 79.0 | Healthy participants (WI, MIHT, PHQ-D) | Signal detection task for NSCF | *d’; c* | 4 | No significant correlations of IAcc x questionnaire data | Significant correlations of RB x WI and MIHT perceptive subscale | | |  |
|  |  |  |  |  | Heartbeat mental tracking task (2) | Relative difference between actual and counted heartbeats |  | Significant negative correlation of IAcc x WI and MIHT affective subscale |  | | |  |
| Krautwurst, Gerlach, and Witthöft (2016) | EG: 40  CG: 41 | NA | NA | Illness anxiety disorder (criteria by 4; WI score > 7) | Signal detection task for NSCF | *d’; c* | 2 | EG > CG | EG < CG | | | Including the diagnosis of an anxiety disorder as a covariate rendered group differences non-significant. No significant of this covariate on IAcc |
|  | EG: 49  CG: 56 |  |  |  | Heartbeat mental tracking task (2) | Relative difference between actual and counted heartbeats |  | EG = CG |  | | | Including the diagnosis of an anxiety disorder as a covariate rendered group differences non-significant. No significant of this covariate on RB |
| Lee et al. (2018) | EG: 23  CG: 20 | EG: *M* = 35 (*SD* = 10.6)  CG: *M* = 30.6 (*SD =* 8.1) | EG: 73.9  CG: 70.0 | Somatic symptom disorder (DSM-5) | Heartbeat perception task (Ehlers & Breuer, 1992) | Heartbeat estimation error | 1 | EG < CG |  | | |  |
| Meyerholz, Irzinger, Witthöft, Gerlach, and Pohl (2019) | 100 | *M* = 25.28 (*SD* = 5.67) | 74.0 | Healthy participants (PHQ-D) | Heartbeat mental tracking task (2) | Relative difference between actual and counted heartbeats |  | No significant correlation of IAcc x PHQ-D |  | | |  |
| Miles, Poliakoff, and Brown (2011) | EG: 20 CG: 20 | EG: *M* = 19.6 (*SD* = 1.5)  CG: *M* = 19.8 (*SD* = 1.6) | EG: 85.0  CG: 70.0. | High vs. low symptom reporters (SDQ) | Rubber hand illusion paradigm | Proprioceptive drift (synchronous condition) | 1 | EG > CG |  | | |  |
| Perepelkina, Romanov, Arina, Volel, and Nikolaeva (2019) | EG: 16  CG: 17 | EG: *M* = 37.1 (*SD* = 10.1)  CG: *M* = 30.2 (*SD* = 3.8) | EG: 56.3  CG: 70.6 | Somatoform disorder (ICD-10) | Rubber hand illusion paradigm | Perceptual drift, onset stage | 10 | EG = CG |  | | | Higher increase of proprioceptive drift during the illusion onset stage in the CG vs. EG |
|  |  |  |  |  | Virtual hand illusion paradigm | Perceptual drift, onset stage |  | EG = CG |  | | | Similar time dynamics of proprioceptive drift during the illusion for both groups |
| Petersen, Van Staeyen, Vogele, von Leupoldt, and Van den Bergh (2015) | EG: 25  CG: 25 | Whole sample: *M* = 21.04 (*SD* = 1.8) | 100.0 | High vs. low symptom reporters (CSD) | Breathing resistance task | *d’; c* | 1 | EG = CG | Significant interaction of group x classification for category A: EG showed a more pronounced change from a conservative to a liberal response criterion than CG | | |  |
| Pollatos et al. (2015) | EG: 23  CG: 23 | EG: *M* = 39.9 (*SD* = 8.8)  CG: *M* = 36.5 (*SD* = 7.2) | EG: 78.3 | Multisomatoform disorder (5), undifferentiated somatoform disorder, somatoform disorder not otherwise specified, somatization disorder, somatoform autonomic dysfunction (ICD-10) | Heartbeat mental tracking task (2) | Relative difference between actual and counted number of heartbeats | 1 | EG < CG |  | | | No significant correlation of IAcc x SSAS |
| Rodic, Meyer, Lieb, and Meinlschmidt (2016) | 179 | Range 18-40 | 50.2 | Healthy participants (SOMS, WI) | Vibrotactile perception task | Tactile thresholds | 2 | Significant negative correlation of IAcc x WI, but not SOMS |  | | |  |
| Sachse (1994) | EG: 33  CG: 33 | EG: *M* = 34.7, range 20-67  CG: *M* = 33.5, range 18-71 | EG: 72.7  CG: 72.7 | Psychological Factors Affecting Other Medical Conditions (DSM-III-R) | Heartbeat tracking task (6) | Relative difference between actual and counted number of heartbeats | 2 | EG < CG |  | | | Significant positive correlation for IAcc x subjective rating of performance for the heartbeat mental tracking in the CG |
|  |  |  |  |  | Heartbeat mental tracking task (2) | Relative difference between actual and counted number of heartbeats |  | EG < CG |  | | |  |
| Sarnoch, Adler, and Scholz (1997) | EG: 7  CG: 6 | Whole sample: *M* = 23.8 (*SD* = 2.7) | 100.0 | High vs. low symptom reporters (SOMS) | Muscle tension perception task | *P(A)* | 1 | EG < CG | EG = CG | | |  |
| Schäfer, Egloff, and Witthöft (2012) | EG: 23  CG: 27 | EG: *M* = 45.3 (*SD* = 13.6)  CG: *M* = 41.7 (*SD* = 12.5) | EG: 69.7  CG: 59.3 | Somatization disorder, pain disorder, undifferentiated somatoform disorder (DSM-IV), medically unexplained symptoms | Heartbeat mental tracking task (2) | Relative difference between actual and counted number of heartbeats | 2 | EG = CG |  | | | EG: negative correlation for IAcc x Somatization Index |
|  |  |  |  |  | Heartbeat discrimination task (modified from 7) | *d’* |  | EG = CG | EG < CG | | | EG/CG: positive correlation for IAcc x WI |
| Scholz, Ott, and Sarnoch (2001) | EG: 20  CG: 20 | EG: *M* = 46.0 (*SD* = 7.9)  CG: *M* = 41.8 (*SD* = 8.3) | EG: 35.0  CG: 35.0 | Somatoform disorder (ICD-10) | Muscle tension perception | Perception score (linear regression with subjective rating as dependent and logarithmic EMG score as independent variable) | 1 | EG > CG |  | | |  |
| Schonecke (1995) | EG: 19  CG: 30 | EG: *M* = 33.5 | EG: 52.6 | Functional cardiac disorder | Heartbeat discrimination task (8) | *P(A)* | 1 | EG < CG |  | | |  |
| Schröder, Gerlach, Achenbach, and Martin (2015) | EG: 38  CG: 52 | EG: *M* = 50.9 (*SD* = 9.7)  CG: *M* = 49.1 (*SD* = 9.5) | EG: 47.4  CG: 61.5 | Noncardiac chest pain (distressing chest pain but normal/near normal coronary arteries) | Heartbeat mental tracking task (2) | Relative difference between actual and counted heartbeats | 2 | EG = CG |  | | | EG: positive correlation of chest pain impairment x IAcc |
|  | EG: 41  CG: 51 | EG: *M* = 51.7 (*SD* = 10.5)  CG: *M* = 49.1 (*SD* = 9.6) | EG: 46.3  CG: 62.7 |  | Heartbeat discrimination task (modified after 1) | *d; c* |  | EG = CG | EG = CG | | | EG: Significant negative correlation of self-reported maximum chest pain intensity x RB |
| Schulz et al. (2020) | EG: 29  CG: 29 | EG: *M* = 28.4 (*SD* = 9.7)  CG: *M* = 26 (*SD* = 6.8) | EG: 27.6  CG: 10.4 | High vs. low symptom reporters (SOMS-2) | Heartbeat mental tracking task (2) | Heartbeat Perception Score | 2 | EG = CG |  | | | Negative correlation of heart rate x IAcc |
|  |  |  |  |  | Heartbeat Discrimination Task | *d’* |  |  |  |  |  |  |
| Weiss, Sack, Henningsen, and Pollatos (2014) | EG: 30  CG: 30 | EG: *M* = 44.0 (*SD* = 11.3)  CG: *M* = 39.2 (*SD* = 9.6) | EG: 86.7 | Multisomatoform disorder (5; ICD-10) | Heartbeat mental tracking task (2) | Relative difference between actual and counted heartbeats | 1 | EG < CG |  | | | EG: Positive correlation of pain tolerance x IAcc  CG: Negative correlation of pain threshold and pain tolerance x IAcc |
| Witthöft et al. (2020) | 316 | *M* = 21.2 (*SD* = 2.3) | 51.0 | Healthy participants (PHQ-15) | Heartbeat mental tracking task (2) | Relative difference between actual and counted heartbeats |  | N.s. correlation of IAcc x PHQ-15 |  | | |  |
| Functional syndromes | | | | | | | | | | | | |
| Akyol, Ulus, Tander, Bilgici, and Kuru (2013) | EG: 60  CG: 45 | EG: *M* = 40.2 (*SD* = 8.8)  CG: *M* = 36.6 (*SD* = 9.4) | 100.0 | Fibromyalgia (9) | Knee repositioning task | Repositioning error^f^ | 2 | EG = CG |  | |  | |
| Anastasopoulos et al. (1997) | EG: 25  CG: 26 | EG: *M* = 49.6 (*SD* = 16.2)  CG: *M* = 47.7 (*SD* = 18.0) | NA | Spasmodic torticollis (10) | Subjective vertical | Entry points for signaling subjective verticality; directional bias | 3 | EG < CG for “roll” and “pitch”;  EG = CG for “barbecue” movement |  | EG = CG for directional bias | | |
| Bara-Jimenez, Shelton, Sanger, and Hallett (2000) | EG: 14  CG: 13 | EG: *M* = 50.4 (*SD* = 10.2) | NA | Focal dystonia of the right hand (10) | Somatosensory temporal discrimination task | Tactile temporal threshold | 1 | EG < CG |  | Significant regression of temporal threshold against severity score | | |
| Bara-Jimenez, Shelton, and Hallett (2000) | EG: 17  CG: 13 | EG: *M* = 50.3 (*SD* = 11.6)  CG: *M* = 50.6 (*SD* = 10.4) | NA | Focal dystonia of the right hand (10) | Spatial localization task | Spatial localization error | 2 | EG < CG |  |  | | |
|  |  |  |  |  | Gap detection task | Gap detection threshold |  | EG < CG |  |  | | |
| Bardal. Roeleveld, Ihlen, and Mork (2016) | EG: 25  CG: 25 | EG: *M* = 55.8 (*SD* = 6.8)  CG: *M* = 51.8 (*SD* = 8.3) | 100.0 | Fibromyalgia (9) | Shoulder repositioning task | Repositioning error^f^ | 1 | EG = CG |  |  | | |
| Borg et al. (2018) | EG: 21  CG: 21 | EG: *M* = 50.3 (*SD* = 8.7)  CG: *M* = 46.3 (*SD* = 9.9) | 100.0 | Fibromyalgia (Wolfe, 2011) | Modified heartbeat mental tracking task (Schandry, 1981) | Relative difference between actual and counted heartbeats | 2 | EG = CG |  |  | | |
| Brun, McCabe, and Mercier (2020) | EG: 20  CG: 20 | EG: *M* = 43.1 (*SD* = 15.1)  CG: *M* = 42.9 (*SD* = 12.3) | 100.0 | Fibromyalgia | Arm position matching test | Proprioceptive error | 1 | EG = CG |  |  | | |
| Celenay, Mete, Coban, Oskay, and Erten (2019) | EG: 15  CG: 15 | EG: *M* = 39.7 (*SD* = 10.5)  CG: *M* = 39 (*SD* = 6.9) | 100.0 | Fibromyalgia (Wolfe, 2011) | Trunk repositioning task | Repositioning error^f^ | 1 | EG < CG |  |  | | |
| Cheng, Wang, Lin, Wang, and Lin (2010) | EG: 12  CG: 12 | EG: *M* = 25.4 (*SD* = 2.1)  CG: *M* = 24.9 (*SD* = 1.8) | EG: 50.0  CG: 41.7 | Neck pain | Head repositioning task | Repositioning error^f^ | 2 | EG < CG |  |  | | |
| Demartini et al. (2016) | EG: 20  CG: 20 | EG_1_: *M* = 45.7 (*SD* = 15.8)  EG_2_: *M* = 45.9 (*SD* = 14.8)  CG: *M* = 43.1 (*SD* = 17.0) | EG_1_: 85.0  EG_2_: 75.0  CG: 80.0 | EG_1_: Psychogenic non-epileptic seizures (11) , EG_2_: functional motor symptoms (12) | Heartbeat mental tracking task (2) | Relative difference between actual and counted heartbeats | 2 | EG_1_ = CG  EG_2_ = CG |  |  | | |
| Demartini et al. (2017) | EG: 20  CG: 20 | EG: *M* = 45.75 (*SD* = 15.87)  CG: *M* = 42.10 (*SD* = 13.34) | EG: 70.0  CG: 80.0 | Functional motor symptoms (12) | Heartbeat mental tracking task (2) | Relative difference between actual and counted heartbeats | 1 | EG = CG |  |  | | |
| De Pauw et al. (2017) | EG: 24  CG: 70 | EG: *M* = 59.2 (*SD* = 13.9)  CG: *M* = 54.5 (*SD* = 16) | EG: 83.3  CG: 52.9 | Cervical dystonia (13) | Head repositioning task | Repositioning error^f^ | 8 | EG < CG |  |  | | |
| De Zoete, Osmotherly, Rivett, & Snodgrass (2020) | EG: 50  CG: 50 | EG: *M* = 35.5; range 24.0-55.3  CG: *M* = 34.5; range 26.0-58.0 | EG: 60.0  CG: 54.0 | Chronic neck pain | Head and trunk repositioning task | Repositioning error^f^ | 2 | EG = CG |  |  | | |
| Dumas et al. (2001) | EG_1_: 24  EG_2_: 16  CG: 17 | EG1: *M* = 44.0 (*SD* = 11.9)  EG2: *M* = 39.0 (*SD* = 12.5)  CG: *M* = 43.0 (*SD* = 14.1) | EG_1_: 79.2  EG_2_: 75.0  CG: 64.7 | EG_1_: Cervicogenic headache, EG_2_:  migraine (14, 15) | Head repositioning task | Repositioning error^f^ | 2 | EG = CG |  |  | | |
| Duschek, Montoro, and del Paso (2017) | EG: 45  CG: 31 | EG: *M* = 49.9 (*SD* = 8.8)  CG: *M* = 47.1 (*SD* = 9.4) | EG: 100.0  CG: 100.0 | Fibromyalgia (9) | Heartbeat mental tracking task (2) | Relative difference between actual and counted heartbeats | 1 | EG < CG |  | Negative correlation of IAcc x FIQ in patients | | |
| Edmondston et al. (2017) | EG: 21  CG: 22 | EG: *M* = 29.0 (*SD* = 7.36)  CG: *M* = 25.7 (*SD* = 5.95) | EG: 37.9  CG: 54.6 | Postural neck pain | Head, neck, and shoulder repositioning task | Repositioning error^f^ | 1 | EG = CG |  |  | | |
| Elsig et al. (2014) | EG: 30  CG: 30 | EG: *M* = 36.9 (*SD =* 13.6)  CG: *M* = 37.2 (*SD* = 13.5) | EG: 83.3  CG: 83.3 | Neck pain | Two-point discrimination test | Tactile discrimination threshold | 3 | EG = CG |  |  | | |
|  |  |  |  |  | Head repositioning task | Root mean square error |  | EG < CG |  | Positive correlation of IAcc x Neck Disability Index | | |
| Fiorio et al. (2007) | EG: 9  CG: 11 | EG: *M* = 42.2 (*SD* = 17.5)  CG: *M* = 36.6 (*SD* = 10.9) | EG: 55.6  CG: 63.6 | DYT1 manifesting dystonia (10) | Somatosensory  temporal discrimination task | Temporal discrimination threshold; temporal order judgement | 4 | EG < CG |  |  | | |
| Fiorio et al. (2008) | EG: 19  CG: 19 | EG: *M* = 61.1 (*SD* = 13.0)  CG: *M* = 65.6 (*SD* = 10.2) | EG: 79.0  CG: 47.4 | Blepharospasm | Somatosensory temporal discrimination task | Temporal discrimination threshold | 1 | EG < CG |  |  | | |
| Fiorio et al. (2011) | EG_1_: 15  EG_2_: 15  CG: 24 | EG_1_: *M* = 45.9 (*SD* = 5.6)  EG_2_: *M* = 49.1 (SD = 6.1)  CG: *M* = 44.0 (*SD* = 9.5) | EG1: 46.7  EG2: 66.7  CG: 50.0 | EG_1_: Focal hand dystonia, EG_2_: non-hand dystonia (16) | Rubber hand illusion paradigm | Proprioceptive drift | 4 | Significant group x stroking condition interaction for IAcc as dependent variable for affected hand: synchronous < asynchronous condition (EG1), synchronous > asynchronous condition (EG2, CG). No significant interaction effect for non-affected hand. |  |  | | |
| Gajdos, Chriszto, and Rigo (2020) | EG: 38  CG: 34^b^ | EG: *M* = 21.7 (*SD* = 2.9)  CG: *M* = 22.1 (*SD* = 2.5)^b^ | EG: 55.9  CG: 71.1^b^ | High vs. low gastrointestinal symptom reporters (WGO-IBS) | Heartbeat mental tracking task (Schandry 1981) | Relative difference between actual and counted heartbeats | 1 | EG = CG |  | Positive correlation of IAcc x self-reported accuracy only in the low symptom group | | |
| Goncalves and Silva (2019) | EG: 33  CG: 33 | EG: *M* = 43.6 (*SD* = 13.3)  CG: *M* = 43.5 (*SD* = 14.1) | EG: 78.8  CG: 78.8 | Chronic neck pain | Head repositioning to neutral, head repositioning to 30° rotation, torsion test, figure of eight relocation test | Repositioning error^f^ | 16 | EG < CG |  | Low positive correlations (most < .3) between proprioceptive tests and disability, fear of movement, and catastrophizing in participants with neck pain | | |
| Grip, Sundelin, Gerdle, and Karlsson (2007) | EG: 21  CG: 24 | EG: *M* = 49.0 (*SD* = 16.0)  CG: *M* = 50.0 (*SD* = 18.0) | EG: 66.7  CG: 66.7 | Neck pain (17) | Head repositioning task | Repositioning error^f^ | 4 | EG = CG |  | Positive correlations of constant error in flexion condition x reported perceived health, pain intensity regarding neck and shoulder, and disability (visual analogue scales) | | |
| Jungilligens et al. (2020) | EG: 20  CG: 20 | EG: *M* = 32.9 (*SD* = 12.8)  CG: *M* = 29.4 (*SD* = 9.9) | EG: 70.0  CG: 70.0 | Dissociative seizures (18) | Heartbeat mental tracking task (Schandry 1981) | Relative difference between actual and counted heartbeats | 1 | EG = CG |  |  | | |
| Katschnig et al. (2010) | EG: 11  CG: 10 | EG: *M* = 38.8 (*SD* = 13.8)  CG: *M* = 40.5 (*SD* = 15.6) | EG: 100.0  CG: 100.0 | Fixed Dystonia (19) | Somatosensory temporal discrimination task | Tactile discrimination threshold | 2 | EG = CG |  |  | | |
|  |  |  |  |  |  | Temporal order judgment |  | EG = CG |  |  |  |  |
| Koreki et al. (2020) | EG: 41  CG: 30 | EG: *M* = 32.0 (*SE* = 2.0)  CG: *M* = 32.0 (*SE* = 2.0) | EG: 90.0  CG: 95.1 | Functional seizures (11) | Heartbeat mental tracking task (Schandry 1981) | Relative difference between actual and counted heartbeats | 2 | EG < CG |  |  | | |
|  |  |  |  |  | Heartbeat discrimination task (20) | Ratio of correct to incorrect synchronicity judgments |  | EG = CG |  |  | | |
| Kristjansson, Dall’Alba, and Jull (2003) | EG: 20  CG: 21 | EG: *M* = 30.0 (*SD* = 9.1)  CG: *M* = 26.9 (*SD* = 6.4) | EG: 45.0  CG: 52.4 | Neck pain | Head repositioning task | Repositioning error^f^ | 5 | EG < CG for repositioning to neutral head position  EG = CG for repositioning to 30° rotation position |  |  | | |
|  |  |  |  |  | Preset trunk rotation task |  |  | EG = CG |  |  | | |
|  |  |  |  |  | Figure-of-eight movement task |  |  | EG = CG |  |  | | |
| Lee, Wang, Yao, and Wang (2008) | EG_1_: 39  EG_2_: 44  CG: 44 | *M* = 41.8 (*SD* = 8.5) | 70.1 | Non-clinical daily (EG_1_) and weekly (EG_2_) neck pain | Head repositioning task | Repositioning error^f^ | 8 | Repositioning to neutral head position: EG_1_ and EG_2_ < CG for right and left rotation; EG_1_ < CG and EG_2_ = CG for extension, EG_1_ and EG_2_ = CG for flexion.  Repositioning to target position:  EG_1_ < CG and EG_2_ = CG for left rotation,  EG_1_ and EG_2_ = CG for right rotation, extension and flexion |  | | |  |
| Marinelli et al. (2011) | EG: 10  CG: 10 | EG: *M* = 50.5 (*SD* = 10.1)  CG: *M* = 46.4 (*SD* = 12.6) | EG: 50.0  CG: 60.0 | Cervical Dystonia (TWSTRS) | Reaching movement task | Directional error | 2 | EG < CG for lateral position; EG = CG for central position |  |  | | |
| Morgante et al. (2011) | EG_1_: 10  EG_2_: 10  CG: 16 | EG_1_: *M* = 43.5 (*SD* = 12.0)  EG_2_: *M* = 38.1 (*SD* = 10.2)  CG: *M* = 36.0 (*SD* = 16.2) | EG^1^: 60.0  EG^2^: 60.0  CG: 56.3 | EG1: Primary torsion dystonia, EG^2^: psychogenic dystonia (21) | Somatosensory temporal discrimination task | Temporal discrimination threshold | 4 | EG < CG |  |  | | |
| Nijs, Aerts, and De Meirleir (2006) | EG: 68  CG: 69 | EG: *M* = 38.4 (*SD* = 10.0)  CG: *M* = 37.9 (*SD* = 10.2) | EG: 82.4  CG: 82.6 | Chronic fatigue syndrome (22) | Leg repositioning task | Repositioning error^f^ | 1 | EG = CG |  |  | | |
| Pick et al. (2020) | EG: 19  CG: 20 | EG: *Mdn* = 44.0 (*IQR* = 20.0)  CG: *Mdn* = 27.0 (*IQR* = 9.8) | EG: 79.0  CG: 80.0 | Functional neurological disorder | Heartbeat mental tracking task (Schandry 1981) | Relative difference between actual and counted heartbeats at baseline | 1 | EG = CG |  |  | | |
| Pinsault, Vuillerme, and Pavan (2008) | EG: 7  CG: 7 | EG: *M* = 56.0 (*SD* = 9.0)  CG: *M* = 64.0 (*SD* = 12.0) | EG: 57.1  CG: 42.9 | Neck pain | Head repositioning task | Repositioning error^f^ | 1 | EG < CG |  |  | | |
| Ricciardi et al. (2016) | EG: 16  CG: 17 | EG: *Mdn =* 41.5 (*IQR* = 14.5)  CG: *Mdn* = 33.0 (IQR = 13.0) | EG: 75.0  CG: 70.6 | Functional movement disorder (21) | Heartbeat mental tracking task | Relative difference between actual and counted heartbeats | 1 | EG < CG |  |  | | |
| Rost, Van Ryckeghem, Schulz, Crombez, and Vogele (2017) | EG: 47  CG: 45 | EG: *M* = 45.5 (*SD =* 9.2)  CG: *M* = 44.9 (*SD* = 12.2) | EG: 83.0  CG: 82.2 | Fibromyalgia (Wolfe, 2010) | Heartbeat mental tracking task | Relative difference between actual and counted heartbeats | 1 | EG = CG |  |  | | |
| Sanger, Tarsy, and Pascual-Leone (2001) | EG: 9  CG: 10 | EG: *M* = 57.0 (*SD* = 11.0)  CG: *M* = 48.0 (*SD* = 17.0) | NA | Focal hand dystonia (10) | Somatosensory temporal discrimination task | Temporal discrimination threshold | 2 | EG < CG |  |  | | |
|  |  |  |  |  | Grating orientation task | Spatial discrimination threshold |  | EG < CG |  |  | | |
| Scontrini et al. (2009) | EG: 82  CG: 61 | EG_1_: *M* = 67.7 (*SD* = 1.7)  EG_2_: *M* = 55.6 (*SD* = 2.4)  EG_3_: *M* = 56.7 (*SD* = 5.3)  EG_4_: *M* = 50.4 (*SD* = 5.7)  CG_1_: *M* = 65.0 (*SD* = 1.7)  CG_2_: *M* = 55.0 (*SD* = 2.3)  CG_3_: *M* = 56.0 (*SD* = 5.1)  CG_4_: *M* = 52.6 (*SD* = 4.9) | EG_1_: 68.6  EG_2_: 73.3  EG_3_: 12.5  EG_4_: 55.6  CG_1_: 45.7  CG_2_: 53.5  CG_3_: 50  CG_4_: 55.6 | Focal dystonia: blepharospasm, cervical dystonia (TWSTRS), hand dystonia (10), laryngeal dystonia) | Somatosensory temporal discrimination task | Temporal discrimination threshold | 12 | EG < CG |  |  | | |
| Sjölander, Michaelson, Jaric, and Djupsjobacka (2008) | EG: 9  CG: 16 | EG: *M* = 40.0 (*SD =* 9.0)  CG: *M* = 41.0 (*SD* = 9.0) | EG: 100.0  CG: 81.3 | Neck pain (17) | Head repositioning task | Repositioning error^f^ | 2 | EG = CG |  |  | | |
| Tinazzi, Frasson, Bertolasi, Fiaschi, and Aglioti (1999) | EG: 7  CG: 9 | EG: *M* = 39.5, range 21-58  CG: *M* = 37.2, range 26-60 | EG: 57.1  CG: 55.6 | Generalized dystonia, hand dystonia, segmental dystonia involving the right arm and trunk (16) | Somatosensory temporal discrimination task | Temporal discrimination threshold | 1 | EG < CG |  |  | | |
| Tinazzi et al. (2002) | EG: 10  CG: 12 | EG: *M* = 39.2, range 21-58  CG: *M* = 38.0, range 26-60 | EG: 50.0.  CG: 50.0 | Generalized dystonia, hand dystonia, and segmental dystonia involving the right arm and trunk (10) | Somatosensory temporal discrimination task | Temporal discrimination threshold | 1 | EG < CG |  |  | | |
| Ulus, Akyol, Tander, Bilgici, and Kuru (2013) | EG: 30  CG: 30 | EG: *M* = 41.0 (*SD* = 9.2)  CG: *M* = 40.7 (*SD* = 9.0) | NA | Fibromyalgia (9) | Knee repositioning task | Repositioning error^f^ | 2 | EG = CG |  |  | | |
| Valenzuela-Moguillansky, Reyes-Reyes, and Gaete (2017) | EG: 30  CG: 29 | EG: *M* = 46.8 (*SD* = 12.7)  CG: *M* = 43.52 (*SD* = 11.0) | 100.0. | Fibromyalgia (Wolfe et al., 2010) | Heartbeat Detection Task (Tsakiris et al., 2011) | Heartbeat perception score | 1 | EG = CG |  | Negative correlation between IAcc x passability ratio, a measure of exteroceptive body awareness (Body-Scaled Action Task, 23) | | |
| Woodhouse and Vasseljen (2008) | EG: 57  CG: 57 | EG: *M* = 43.7 (*SD* = 12.6)  CG: *M* = 38.2 (*SD* = 10.9) | EG: 66.7  CG: 49.1 | Chronic non-traumatic neck pain | Head repositioning task | Repositioning error^f^ | 1 | EG = CG |  |  | | |

**References**

1. Brener J, Kluvitse C. Heartbeat detection: judgments of the simultaneity of external stimuli and heartbeats. Psychophysiology. 1988;25(5):554-61.

2. Schandry R. Heart beat perception and emotional experience. Psychophysiology. 1981;18(4):483-8.

3. Katzer A, Oberfeld D, Hiller W, Witthöft M. Tactile perceptual processes and their relationship to medically unexplained symptoms and health anxiety. J Psychosom Res. 2011;71(5):335-41.

4. Fink P, Ornbol E, Toft T, Sparle KC, Frostholm L, Olesen F. A new, empirically established hypochondriasis diagnosis. The American journal of psychiatry. 2004;161(9):1680-91.

5. Kroenke K, Spitzer RL, deGruy FV, 3rd, Hahn SR, Linzer M, Williams JB, et al. Multisomatoform disorder. An alternative to undifferentiated somatoform disorder for the somatizing patient in primary care. Arch Gen Psychiatry. 1997;54(4):352-8.

6. McFarland RA, Campbell C. Precise heart-rate control and heart-rate perception. Percept Mot Skills. 1975;41(3):730.

7. Knoll JF, Hodapp V. A comparison between two methods for assessing heartbeat perception. Psychophysiology. 1992;29(2):218-22.

8. Katkin ES, Blascovich J, Goldband S. Empirical assessment of visceral self-perception: individual and sex differences in the acquisition of heartbeat discrimination. J Pers Soc Psychol. 1981;40(6):1095-101.

9. Wolfe F, Smythe HA, Yunus MB, Bennett RM, Bombardier C, Goldenberg DL, et al. The American College of Rheumatology 1990 Criteria for the Classification of Fibromyalgia. Report of the Multicenter Criteria Committee. Arthritis Rheum. 1990;33(2):160-72.

10. Burke RE, Fahn S, Marsden CD, Bressman SB, Moskowitz C, Friedman J. Validity and reliability of a rating scale for the primary torsion dystonias. Neurology. 1985;35(1):73-7.

11. LaFrance WC, Jr., Baker GA, Duncan R, Goldstein LH, Reuber M. Minimum requirements for the diagnosis of psychogenic nonepileptic seizures: a staged approach: a report from the International League Against Epilepsy Nonepileptic Seizures Task Force. Epilepsia. 2013;54(11):2005-18.

12. Williams DT, Ford B, Fahn S. Phenomenology and psychopathology related to psychogenic movement disorders. Adv Neurol. 1995;65:231-57.

13. Albanese A, Asmus F, Bhatia KP, Elia AE, Elibol B, Filippini G, et al. EFNS guidelines on diagnosis and treatment of primary dystonias. Eur J Neurol. 2011;18(1):5-18.

14. Sjaastad O, Frederiksen TA. Chronic daily headache: is "cervicogenic headache" one subgroup? Cephalalgia. 1998;18:37-40.

15. International Headache Society. Classification and diagnostic criteria for headache disorders, cranial neuralgias and facial pain. Headache Classification Committee of the International Headache Society. Cephalalgia. 1988;8 Suppl 7:1-96.

16. Fahn S. Assessment of the primary dystonia. In: Munsat TL, editor. Quantification of Neurological Deficit. Stoneham: Butterworths; 1989.

17. Spitzer WO, Skooron ML, Salmi LR, Cassidy JD, Duranceau J, Suissa S, et al. Scientific Monograph of the Quebec Task-Force on Whiplash-Associated Disorders - Redefining Whiplash and Its Management (Vol 20, Pg 1, Pg 1995). Spine. 1995;20(21):2372-.

18. Popkirov S, Jungilligens J, Grönheit W, Wellmer J. Diagnosing psychogenic nonepileptic seizures: Video-EEG monitoring, suggestive seizure induction and diagnostic certainty. Epilepsy Behav. 2017;73:54-8.

19. Schrag A, Trimble M, Quinn N, Bhatia K. The syndrome of fixed dystonia: an evaluation of 103 patients. Brain. 2004;127(Pt 10):2360-72.

20. Whitehead WE, Drescher VM, Heiman P, Blackwell B. Relation of heart rate control to heartbeat perception. Biofeedback and Self-regulation. 1977;2(4):371-92.

21. Fahn S, Williams DT. Psychogenic dystonia. Adv Neurol. 1988;50:431-55.

22. Fukuda K, Straus SE, Hickie I, Sharpe MC, Dobbins JG, Komaroff A. The chronic fatigue syndrome: a comprehensive approach to its definition and study. International Chronic Fatigue Syndrome Study Group. Ann Intern Med. 1994;121(12):953-9.

23. Guardia D, Lafargue G, Thomas P, Dodin V, Cottencin O, Luyat M. Anticipation of body-scaled action is modified in anorexia nervosa. Neuropsychologia. 2010;48(13):3961-6.
