# Supplementary material for: Interoceptive accuracy and bias in somatic symptom disorder, illness anxiety disorder, and functional syndromes: A systematic review and meta-analysis
Source: PLoS One. 2022 Aug 18;17(8):e0271717. doi: 10.1371/journal.pone.0271717 (PMC9387777; doi:10.1371/journal.pone.0271717)
Supplement: S3 Table — (DOCX) [file pone.0271717.s004.docx]

**S3 Table**

| **Task** | **Description** | **Outcome measure** | **Accuracy separated from bias** | **Reference for task protocol** | **References for studies from meta-analysis** |
| --- | --- | --- | --- | --- | --- |
| Mental tracking task | | | | | |
| Mental tracking task | Participants count their heartbeats in different time intervals without feeling their pulse or using any other aids. | Heartbeat perception score: Relative difference between actual and counted heartbeats | No | [1] | [2-25] |
| Signal detection tasks | | | | | |
| Heartbeat discrimination task | Participants rate external cues (tones) as synchronous or asynchronous to their own heartbeat | *d’*, *c*  interquartile ranges | Yes | [26] | [9-11, 20, 27, 28] |
| Somatic signal detection task | Participants are asked to report a vibration on their fingertip as present or absent | Tactile threshold, *c* | Yes | [29] | [30-32] |
| Signal detection task for non-specific skin conductance fluctuations | Participants indicate whether they perceived physiological arousal when an acoustic signal is presented. Sound signals are triggered by either skin conductance arousal or stable skin conductance for 20 s | *d', c* | Yes | [33] | [4, 5] |
| Breathing resistance task | Different breathing resistances have to be memorized and classified afterwards | *d’, c* | Yes | [34] | [34] |
| Muscular signal detection task | Pairs of muscular tension levels have to be produced according to a feedback signal and then judged to be equal or unequal | *P(A), B* | Yes | [35] | [35] |
| Thresholding procedures | | | | |  |
| Threshold detection task | Participants estimate if a tactile stimulus was present | Tactile threshold | No | e.g. [36] | [36] |
| Temporal threshold task | Participants indicate whether they perceived one or two stimuli after consecutive presentation of two tactile stimuli | Tactile temporal threshold | No | e.g. [37] | [37-44] |
| Spatial threshold task | Participants indicate whether they perceived notches in objects as horizontal or vertical lines | Spatial discrimination threshold | No | e.g. [44] | [37, 44] |
| Position sense tasks | | | | | |
| (Re)positioning task | Participants memorize  and reproduce specific  body positions, or  indicate upright body  posture without visual  cues | (Re)positioning error | No | e.g. [45] | [3, 37, 45-64] |
| Reaching movement task | Participants make out and back movements with their arm on a digitizing tablet following target. Targets comprise eight circles placed at 10cm from a central starting point. | Directional error | No | [65] | [65] |
| Rubber and virtual hand task | | | | | |
| Rubber hand task | An illusory perception of the own hand shifted in the direction of a rubber hand is evoked and the distance between the actual hand at its estimated location is measured | Proprioceptive drift | No | [66] | [67-69] |
| Virtual hand illusion | Same as rubber hand task, but using a virtual reality scene | Proprioceptive drift | No | [68] | [68] |
| Correlational tasks | | | | | |
| Muscle tension perception | Participants are instructed to generate a certain muscle tension and rate their perceived tension afterwards | Correlation between perceived and requested tension | No | [70] | [70] |
| Rebreathing task | Participants inhale different type of air mixtures. After each trial they fill out an online rating scale (respiratory sensations or symptoms and complaints). | Correlations between subjective rating and minute ventilation | No | [71] | [71] |

**References**

1. Schandry R. Heart beat perception and emotional experience. Psychophysiology. 1981;18(4):483-8. doi: 10.1111/j.1469-8986.1981.tb02486.x. PubMed PMID: 7267933.

2. Bräscher AK, Schulz SM, Van den Bergh O, Witthöft M. Prospective study of nocebo effects related to symptoms of idiopathic environmental intolerance attributed to electromagnetic fields (IEI-EMF). Environ Res. 2020;190. doi: ARTN 110019

10.1016/j.envres.2020.110019. PubMed PMID: WOS:000576671000001.

3. Ferentzi E, Bogdany T, Szabolcs Z, Csala B, Horvath A, Koteles F. Multichannel Investigation of Interoception: Sensitivity Is Not a Generalizable Feature. Front Hum Neurosci. 2018;12:223. Epub 2018/06/19. doi: 10.3389/fnhum.2018.00223. PubMed PMID: 29910718; PubMed Central PMCID: PMCPMC5992275.

4. Krautwurst S, Gerlach AL, Gomille L, Hiller W, Witthöft M. Health anxiety - An indicator of higher interoceptive sensitivity? J Behav Ther Exp Psy. 2014;45(2):303-9. doi: 10.1016/j.jbtep.2014.02.001. PubMed PMID: WOS:000334013400012.

5. Krautwurst S, Gerlach AL, Witthöft M. Interoception in pathological health anxiety. J Abnorm Psychol. 2016;125(8):1179-84. doi: 10.1037/abn0000210.

6. Lee D, Kim SJ, Cheon J, Hwang EH, Jung YC, Kang JI. Characteristics of Autonomic Activity and Reactivity During Rest and Emotional Processing and Their Clinical Correlations in Somatic Symptom Disorder. Psychosomatic Medicine. 2018;80(8):690-7. doi: 10.1097/Psy.0000000000000622. PubMed PMID: WOS:000447173000001.

7. Meyerholz L, Irzinger J, Witthöft M, Gerlach AL, Pohl A. Contingent biofeedback outperforms other methods to enhance the accuracy of cardiac interoception: A comparison of short interventions. J Behav Ther Exp Psychiatry. 2019;63:12-20. Epub 2018/12/18. doi: 10.1016/j.jbtep.2018.12.002. PubMed PMID: 30557753.

8. Sachse R. [Heart beat perception in patients with psychosomatic disorders: distracting attention from personal physical processes]. Psychother Psychosom Med Psychol. 1994;44(8):284-92. Epub 1994/08/01. PubMed PMID: 7938376.

9. Schäfer M, Egloff B, Witthöft M. Is Interoceptive Awareness Really Altered in Somatoform Disorders? Testing Competing Theories With Two Paradigms of Heartbeat Perception. J Abnorm Psychol. 2012;121(3):719-24. doi: 10.1037/a0028509. PubMed PMID: WOS:000307482700018.

10. Schröder S, Gerlach AL, Achenbach S, Martin A. The Relevance of Accuracy of Heartbeat Perception in Noncardiac and Cardiac Chest Pain. Int J Behav Med. 2015;22(2):258-67. doi: 10.1007/s12529-014-9433-3. PubMed PMID: WOS:000351523600012.

11. Schulz A, Rost S, Flasinski T, Dierolf AM, Lutz APC, Munch EE, et al. Distinctive body perception mechanisms in high versus low symptom reporters: A neurophysiological model for medically-unexplained symptoms. J Psychosom Res. 2020;137:110223. Epub 2020/09/01. doi: 10.1016/j.jpsychores.2020.110223. PubMed PMID: 32866840.

12. Weiss S, Sack M, Henningsen P, Pollatos O. On the Interaction of Self-Regulation, Interoception and Pain Perception. Psychopathology. 2014;47(6):377-82. doi: 10.1159/000365107. PubMed PMID: WOS:000345661400005.

13. Witthöft M, Bräscher AK, Jungmann SM, Koteles F. Somatic Symptom Perception and Interoception A Latent-Variable Approach. Z Psychol. 2020;228(2):100-9. doi: 10.1027/2151-2604/a000403. PubMed PMID: WOS:000535722000005.

14. Borg C, Chouchou F, Dayot-Gorlero J, Zimmerman P, Maudoux D, Laurent B, et al. Pain and emotion as predictive factors of interoception in fibromyalgia. J Pain Res. 2018;11:823-35. doi: 10.2147/Jpr.S152012. PubMed PMID: WOS:000431255700001.

15. Demartini B, Goeta D, Barbieri V, Ricciardi L, Canevini MP, Turner K, et al. Psychogenic non-epileptic seizures and functional motor symptoms: A common phenomenology? J Neurol Sci. 2016;368:49-54. Epub 2016/08/20. doi: 10.1016/j.jns.2016.06.045. PubMed PMID: 27538601.

16. Demartini B, Goeta D, Romito L, Anselmetti S, Bertelli S, D'Agostino A, et al. Anorexia Nervosa and Functional Motor Symptoms: Two Faces of the Same Coin? J Neuropsychiatry Clin Neurosci. 2017;29(4):383-90. Epub 2017/06/01. doi: 10.1176/appi.neuropsych.16080156. PubMed PMID: 28558480.

17. Duschek S, Montoro CI, del Paso GAR. Diminished Interoceptive Awareness in Fibromyalgia Syndrome. Behav Med. 2017;43(2):100-7. doi: 10.1080/08964289.2015.1094442. PubMed PMID: WOS:000402671700003.

18. Gajdos P, Chriszto Z, Rigo A. The association of different interoceptive dimensions with functional gastrointestinal symptoms. J Health Psychol. 2020:1359105320929426. Epub 2020/06/17. doi: 10.1177/1359105320929426. PubMed PMID: 32538172.

19. Jungilligens J, Wellmer J, Schlegel U, Kessler H, Axmacher N, Popkirov S. Impaired emotional and behavioural awareness and control in patients with dissociative seizures. Psychol Med. 2020;50(16):2731-9. Epub 2019/10/19. doi: 10.1017/S0033291719002861. PubMed PMID: 31625504.

20. Koreki A, Garfkinel SN, Mula M, Agrawal N, Cope S, Eilon T, et al. Trait and state interoceptive abnormalities are associated with dissociation and seizure frequency in patients with functional seizures. Epilepsia. 2020;61(6):1156-65. Epub 2020/06/06. doi: 10.1111/epi.16532. PubMed PMID: 32501547; PubMed Central PMCID: PMCPMC7737228.

21. Pick S, Rojas-Aguiluz M, Butler M, Mulrenan H, Nicholson TR, Goldstein LH. Dissociation and interoception in functional neurological disorder. Cogn Neuropsychiatry. 2020;25(4):294-311. Epub 2020/07/09. doi: 10.1080/13546805.2020.1791061. PubMed PMID: 32635804.

22. Ricciardi L, Demartini B, Crucianelli L, Krahe C, Edwards MJ, Fotopoulou A. Interoceptive awareness in patients with functional neurological symptoms. Biol Psychol. 2016;113:68-74. Epub 2015/11/04. doi: 10.1016/j.biopsycho.2015.10.009. PubMed PMID: 26528552.

23. Rost S, Van Ryckeghem DM, Schulz A, Crombez G, Vogele C. Generalized hypervigilance in fibromyalgia: Normal interoceptive accuracy, but reduced self-regulatory capacity. J Psychosom Res. 2017;93:48-54. Epub 2017/01/22. doi: 10.1016/j.jpsychores.2016.12.003. PubMed PMID: 28107892.

24. Valenzuela-Moguillansky C, Reyes-Reyes A, Gaete MI. Exteroceptive and Interoceptive Body-Self Awareness in Fibromyalgia Patients. Front Hum Neurosci. 2017;11:117. Epub 2017/03/30. doi: 10.3389/fnhum.2017.00117. PubMed PMID: 28348526; PubMed Central PMCID: PMCPMC5346579.

25. Pollatos O, Dietel A, Herbert BM, Wankner S, Wachsmuth C, Henningsen P, et al. Blunted autonomic reactivity and increased pain tolerance in somatoform patients. Pain. 2011;152(9):2157-64. Epub 2011/06/24. doi: 10.1016/j.pain.2011.05.024. PubMed PMID: 21696888.

26. Brener J, Kluvitse C. Heartbeat detection: judgments of the simultaneity of external stimuli and heartbeats. Psychophysiology. 1988;25(5):554-61. Epub 1988/09/01. doi: 10.1111/j.1469-8986.1988.tb01891.x. PubMed PMID: 3186884.

27. Barsky AJ, Brener J, Coeytaux RR, Cleary PD. Accurate awareness of heartbeat in hypochondriacal and non-hypochondriacal patients. J Psychosom Res. 1995;39(4):489-97. Epub 1995/05/01. doi: 10.1016/0022-3999(94)00166-3. PubMed PMID: 7562678.

28. Schonecke OW. Functional cardiac disorder and cardiac perception: Attempts of quantification. In: Schonecke OW, Vaitl D, Schandy R, editors. From the heart to the brain: The psychophysiology of circulation - brain interaction. Frankfurt/Main: Peter Lang-Verlag; 1995.

29. Lloyd DM, Mason L, Brown RJ, Poliakoff E. Development of a paradigm for measuring somatic disturbance in clinical populations with medically unexplained symptoms. J Psychosom Res. 2008;64(1):21-4. doi: 10.1016/j.jpsychores.2007.06.004. PubMed PMID: 18157995.

30. Brown RJ, Brunt N, Poliakoff E, Lloyd DM. Illusory touch and tactile perception in somatoform dissociators. J Psychosom Res. 2010;69(3):241-8. Epub 2010/08/17. doi: 10.1016/j.jpsychores.2009.11.010. PubMed PMID: 20708446.

31. Katzer A, Oberfeld D, Hiller W, Gerlach AL, Witthöft M. Tactile Perceptual Processes and Their Relationship to Somatoform Disorders. J Abnorm Psychol. 2012;121(2):530-43. doi: 10.1037/a0026536. PubMed PMID: WOS:000304131400023.

32. Katzer A, Oberfeld D, Hiller W, Witthöft M. Tactile perceptual processes and their relationship to medically unexplained symptoms and health anxiety. J Psychosom Res. 2011;71(5):335-41. Epub 2011/10/18. doi: 10.1016/j.jpsychores.2011.03.009. PubMed PMID: 21999977.

33. Andor T, Gerlach AL, Rist F. Superior perception of phasic physiological arousal and the detrimental consequences of the conviction to be aroused on worrying and metacognitions in GAD. J Abnorm Psychol. 2008;117(1):193-205. doi: 10.1037/0021-843x.117.1.193. PubMed PMID: WOS:000252990000016.

34. Petersen S, Van Staeyen K, Vogele C, von Leupoldt A, Van den Bergh O. Interoception and symptom reporting: disentangling accuracy and bias. Front Psychol. 2015;6:732. Epub 2015/06/20. doi: 10.3389/fpsyg.2015.00732. PubMed PMID: 26089810; PubMed Central PMCID: PMCPMC4454884.

35. Sarnoch H, Adler F, Scholz OB. Die Wahrnehmung propriozeptiver Reize in Abhängigkeit von der Somatisierungstendenz. Verhaltenstherapie und Verhaltensmedizin. 1997;18:419-28.

36. Rodic D, Meyer AH, Lieb R, Meinlschmidt G. The Association of Sensory Responsiveness with Somatic Symptoms and Illness Anxiety. Int J Behav Med. 2016;23(1):39-48. Epub 2015/04/22. doi: 10.1007/s12529-015-9483-1. PubMed PMID: 25896875.

37. Bara-Jimenez W, Shelton P, Hallett M. Spatial discrimination is abnormal in focal hand dystonia. Neurology. 2000;55(12):1869-73. doi: Doi 10.1212/Wnl.55.12.1869. PubMed PMID: WOS:000166018800020.

38. Fiorio M, Gambarin M, Valente EM, Liberini P, Loi M, Cossu G, et al. Defective temporal processing of sensory stimuli in DYT1 mutation carriers: a new endophenotype of dystonia? Brain. 2007;130:134-42. doi: 10.1093/brain/awl283. PubMed PMID: WOS:000243061500012.

39. Fiorio M, Tinazzi M, Scontrini A, Stanzani C, Gambarin M, Fiaschi A, et al. Tactile temporal discrimination in patients with blepharospasm. J Neurol Neurosurg Psychiatry. 2008;79(7):796-8. Epub 2007/11/08. doi: 10.1136/jnnp.2007.131524. PubMed PMID: 17986501.

40. Morgante F, Tinazzi M, Squintani G, Martino D, Defazio G, Romito L, et al. Abnormal tactile temporal discrimination in psychogenic dystonia. Neurology. 2011;77(12):1191-7. doi: DOI 10.1212/WNL.0b013e31822f0449. PubMed PMID: WOS:000295027800017.

41. Scontrini A, Conte A, Defazio G, Fiorio M, Fabbrini G, Suppa A, et al. Somatosensory temporal discrimination in patients with primary focal dystonia. J Neurol Neurosurg Psychiatry. 2009;80(12):1315-9. Epub 2009/06/23. doi: 10.1136/jnnp.2009.178236. PubMed PMID: 19541688.

42. Tinazzi M, Fiaschi A, Frasson E, Fiorio M, Cortese F, Aglioti SM. Deficits of temporal discrimination in dystonia are independent from the spatial distance between the loci of tactile stimulation. Mov Disord. 2002;17(2):333-8. doi: 10.1002/mds.10019. PubMed PMID: WOS:000174740500015.

43. Tinazzi M, Frasson E, Bertolasi L, Fiaschi A, Aglioti S. Temporal discrimination of somesthetic stimuli is impaired in dystonic patients. Neuroreport. 1999;10(7):1547-50. doi: Doi 10.1097/00001756-199905140-00028. PubMed PMID: WOS:000080653300029.

44. Sanger TD, Tarsy D, Pascual-Leone A. Abnormalities of spatial and temporal sensory discrimination in writer's cramp. Mov Disord. 2001;16(1):94-9. doi: 10.1002/1531-8257(200101)16:1<94::aid-mds1020>3.0.co;2-o. PubMed PMID: WOS:000166762900014.

45. Dumas JP, Arsenault AB, Boudreau G, Magnoux E, Lepage Y, Bellavance A, et al. Physical impairments in cervicogenic headache: traumatic vs. nontraumatic onset. Cephalalgia. 2001;21(9):884-93. doi: DOI 10.1046/j.1468-2982.2001.00264.x. PubMed PMID: WOS:000173046000005.

46. Akyol Y, Ulus Y, Tander B, Bilgici A, Kuru O. Muscle Strength, Fatigue, Functional Capacity, and Proprioceptive Acuity in Patients With Fibromyalgia. Turk Fiz Tip Rehab D. 2013;59(4):292-8. doi: 10.4274/tftr.22230. PubMed PMID: WOS:000340589000005.

47. Anastasopoulos D, Bhatia K, Bisdorff A, Bronstein AM, Gresty MA, Marsden CD. Perception of spatial orientation in spasmodic torticollis .1. The postural vertical. Mov Disord. 1997;12(4):561-9. doi: DOI 10.1002/mds.870120413. PubMed PMID: WOS:A1997XM20000012.

48. Bardal EM, Roeleveld K, Ihlen E, Mork PJ. Micro movements of the upper limb in fibromyalgia: The relation to proprioceptive accuracy and visual feedback. J Electromyogr Kines. 2016;26:1-7. doi: 10.1016/j.jelekin.2015.12.006. PubMed PMID: WOS:000370187700003.

49. Brun C, McCabe CS, Mercier C. The Contribution of Motor Commands to the Perturbations Induced by Sensorimotor Conflicts in Fibromyalgia. Neuroscience. 2020;434:55-65. Epub 2020/03/23. doi: 10.1016/j.neuroscience.2020.03.017. PubMed PMID: 32200078.

50. Celenay ST, Mete O, Coban O, Oskay D, Erten S. Trunk position sense, postural stability, and spine posture in fibromyalgia. Rheumatol Int. 2019;39(12):2087-94. doi: 10.1007/s00296-019-04399-1. PubMed PMID: WOS:000505166800009.

51. Cheng CH, Wang JL, Lin JJ, Wang SF, Lin KH. Position accuracy and electromyographic responses during head reposition in young adults with chronic neck pain. J Electromyogr Kines. 2010;20(5):1014-20. doi: 10.1016/j.jelekin.2009.11.002. PubMed PMID: WOS:000280576100031.

52. De Pauw J, Mercelis R, Hallemans A, Michiels S, Truijen S, Cras P, et al. Cervical sensorimotor control in idiopathic cervical dystonia: A cross-sectional study. Brain Behav. 2017;7(9):e00735. Epub 2017/09/28. doi: 10.1002/brb3.735. PubMed PMID: 28948067; PubMed Central PMCID: PMCPMC5607536.

53. De Zoete RMJ, Osmotherly PG, Rivett DA, Snodgrass SJ. No Differences Between Individuals With Chronic Idiopathic Neck Pain and Asymptomatic Individuals on 7 Cervical Sensorimotor Control Tests: A Cross-sectional Study. J Orthop Sport Phys. 2020;50(1):33-43. doi: 10.2519/jospt.2020.8846. PubMed PMID: WOS:000505052000005.

54. Edmondston SJ, Chan HY, Ngai GC, Warren ML, Williams JM, Glennon S, et al. Postural neck pain: an investigation of habitual sitting posture, perception of 'good' posture and cervicothoracic kinaesthesia. Man Ther. 2007;12(4):363-71. Epub 2006/09/12. doi: 10.1016/j.math.2006.07.007. PubMed PMID: 16963312.

55. Elsig S, Luomajoki H, Sattelmayer M, Taeymans J, Tal-Akabi A, Hilfiker R. Sensorimotor tests, such as movement control and laterality judgment accuracy, in persons with recurrent neck pain and controls. A case-control study. Manual Ther. 2014;19(6):555-61. doi: 10.1016/j.math.2014.05.014. PubMed PMID: WOS:000347721000009.

56. Goncalves C, Silva AG. Reliability, measurement error and construct validity of four proprioceptive tests in patients with chronic idiopathic neck pain. Musculoskelet Sci Pract. 2019;43:103-9. Epub 2019/08/04. doi: 10.1016/j.msksp.2019.07.010. PubMed PMID: 31376618.

57. Grip H, Sundelin G, Gerdle B, Karlsson JS. Variations in the axis of motion during head repositioning - A comparison of subjects with whiplash-associated disorders or non-specific neck pain and healthy controls. Clin Biomech. 2007;22(8):865-73. doi: 10.1016/j.clinbiomech.2007.05.008. PubMed PMID: WOS:000249643200001.

58. Kristjansson E, Dall'Alba P, Jull G. A study of five cervicocephalic relocation tests in three different subject groups. Clin Rehabil. 2003;17(7):768-74. doi: 10.1191/0269215503cr676oa. PubMed PMID: WOS:000186152000011.

59. Lee HY, Wang JD, Yao G, Wang SF. Association between cervicocephalic kinesthetic sensibility and frequency of subclinical neck pain. Manual Ther. 2008;13(5):419-25. doi: 10.1016/j.math.2007.04.001. PubMed PMID: WOS:000259379700007.

60. Nijs J, Aerts A, De Meirleir K. Generalized joint hypermobility is more common in chronic fatigue syndrome than in healthy control subjects. J Manip Physiol Ther. 2006;29(1):32-9. doi: 10.1016/j.jmpt.2005.11.004. PubMed PMID: WOS:000234605000005.

61. Pinsault N, Vuillerme N, Pavan P. Cervicocephalic relocation test to the neutral head position: assessment in bilateral labyrinthine-defective and chronic, nontraumatic neck pain patients. Arch Phys Med Rehabil. 2008;89(12):2375-8. Epub 2008/12/09. doi: 10.1016/j.apmr.2008.06.009. PubMed PMID: 19061750.

62. Sjolander P, Michaelson P, Jaric S, Djupsjobacka M. Sensorimotor disturbances in chronic neck pain--range of motion, peak velocity, smoothness of movement, and repositioning acuity. Man Ther. 2008;13(2):122-31. Epub 2007/01/02. doi: 10.1016/j.math.2006.10.002. PubMed PMID: 17197230.

63. Ulus Y, Akyol Y, Tander B, Bilgici A, Kuru O. Knee Proprioception and Balance in Turkish Women With and Without Fibromyalgia Syndrome. Turk Fiz Tip Rehab D. 2013;59(2):128-32. doi: 10.4274/tftr.75428. PubMed PMID: WOS:000321475100009.

64. Woodhouse A, Vasseljen O. Altered motor control patterns in whiplash and chronic neck pain. Bmc Musculoskel Dis. 2008;9. doi: Artn 90

10.1186/1471-2474-9-90. PubMed PMID: WOS:000257493700001.

65. Marinelli L, Pelosin E, Trompetto C, Avanzino L, Ghilardi MF, Abbruzzese G, et al. In idiopathic cervical dystonia movement direction is inaccurate when reaching in unusual workspaces. Parkinsonism Relat D. 2011;17(6):470-2. doi: 10.1016/j.parkreldis.2011.01.017. PubMed PMID: WOS:000292948500015.

66. Tsakiris M, Tajadura-Jimenez A, Costantini M. Just a heartbeat away from one's body: interoceptive sensitivity predicts malleability of body-representations. P Roy Soc B-Biol Sci. 2011;278(1717):2470-6. doi: 10.1098/rspb.2010.2547. PubMed PMID: WOS:000292592000009.

67. Miles E, Poliakoff E, Brown RJ. Medically unexplained symptom reports are associated with a decreased response to the rubber hand illusion. J Psychosom Res. 2011;71(4):240-4. Epub 2011/09/14. doi: 10.1016/j.jpsychores.2011.04.002. PubMed PMID: 21911101.

68. Perepelkina O, Romanov D, Arina G, Volel B, Nikolaeva V. Multisensory mechanisms of body perception in somatoform disorders. J Psychosom Res. 2019;127. doi: ARTN 109837

10.1016/j.jpsychores.2019.109837. PubMed PMID: WOS:000503320200005.

69. Fiorio M, Weise D, Onal-Hartmann C, Zeller D, Tinazzi M, Classen J. Impairment of the rubber hand illusion in focal hand dystonia. Brain. 2011;134:1428-37. doi: 10.1093/brain/awr026. PubMed PMID: WOS:000290818600022.

70. Scholz OB, Ott R, Sarnoch H. Proprioception in somatoform disorders. Behav Res Ther. 2001;39(12):1429-38. doi: Doi 10.1016/S0005-7967(00)00108-X. PubMed PMID: WOS:000172364700004.

71. Bogaerts K, Millen A, Li W, De Peuter S, Van Diest I, Vlemincx E, et al. High symptom reporters are less interoceptively accurate in a symptom-related context. J Psychosom Res. 2008;65(5):417-24. doi: 10.1016/j.jpsychores.2008.03.019. PubMed PMID: WOS:000260687900003.
