# Supplementary material for: Interoceptive accuracy and bias in somatic symptom disorder, illness anxiety disorder, and functional syndromes: A systematic review and meta-analysis
Source: PLoS One. 2022 Aug 18;17(8):e0271717. doi: 10.1371/journal.pone.0271717 (PMC9387777; doi:10.1371/journal.pone.0271717)
Supplement: S4 Table — Yes (low risk of bias); no (high risk of bias);? (unclear); NA (not applicable). (DOCX) [file pone.0271717.s005.docx]

**S4 Table**

| Authors (Year) | Replicable Sampling Methods | Age matching of controls | | | | Diagnosis of controls | | Any psycho-pathological conditions excluded | Replicable protocol cited or described | Accuracy vs. response bias assessed | No missing data or missing data described | Count “yes” (out of 9) |
| --- | --- | --- | --- | --- | --- | --- | --- | --- | --- | --- | --- | --- |
|  |  | Matched | Statistics provided | Reported in text/table | |  |  |  |  |  |  |  |
| Somatic symptom disorders / illness anxiety disorders | | | | | | | | | | | |  |
| Barsky, Brener, Coeytaux, and Cleary (1995) | Yes | No | Yes | Yes | | Yes | | No | Yes | No | Yes | 6 |
| Bogaerts et al. (2008) | Yes | ? | No | No | | NA | | Yes | Yes | No | No | 3 |
| Bräscher, Schulz, Van den Bergh, and Witthöft (2020) | No | NA | NA | NA | | NA | | Yes | Yes | No | Yes | 3 |
| Brown, Brunt, Poliakoff, and Lloyd (2010) | Yes | Yes | No | Yes | | NA | | No | Yes | Yes | Yes | 5 |
| Ferentzi, Horvath, and Koteles (2019) | No | NA | NA | NA | | NA | | No | Yes | No | Yes | 2 |
| Haenen, Schmidi, Schoenmakers, and van den Hout (1997) | Yes | Yes | No | Yes | | Yes | | No | Yes | No | Yes | 6 |
| Katzer, Oberfeld, Hiller, and Witthöft (2011) | Yes | NA | NA | NA | | NA | | No | Yes | Yes | Yes | 4 |
| Katzer, Oberfeld, Hiller, Gerlach, and Witthöft (2012) | Yes | Yes | Yes | Yes | | Yes | | Yes | Yes | Yes | Yes | 9 |
| Krautwurst, Gerlach, Gomille, Hiller, and Witthöft (2014) | Yes | NA | NA | NA | | NA | | Yes | Yes | Yes | Yes | 5 |
| Krautwurst, Gerlach, and Witthöft (2016) | Yes | Yes | Yes | Yes | | Yes | | Yes | Yes | Yes | Yes | 9 |
| Lee et al. (2018) | Yes | Yes | Yes | Yes | | Yes | | Yes | Yes | No | Yes | 8 |
| Meyerholz, Irzinger, Witthöft, Gerlach, and Pohl (2019) | Yes | NA | NA | NA | | NA | | No | Yes | No | Yes | 3 |
| Miles, Poliakoff, and Brown (2011) | Yes | Yes | No | Yes | | NA | | No | Yes | No | Yes | 5 |
| Perepelkina, Romanov, Arina, Volel, and Nikolaeva (2019) | No | No | No | Yes | | Yes | | Yes | Yes | No | Yes | 5 |
| Petersen, Van Staeyen, Vogele, von Leupoldt, and Van den Bergh (2015) | No | ? | No | No | | NA | | No | Yes | Yes | Yes | 3 |
| Pollatos et al. (2011) | Yes | Yes | Yes | Yes | | Yes | | Yes | Yes | No | No | 7 |
| Rodic, Meyer, Lieb, and Meinlschmidt (2016) | Yes | NA | NA | NA | | NA | | No | Yes | No | Yes | 3 |
| Sachse (1994) | No | ? | No | No | | No | | No | Yes | No | No | 1 |
| Sarnoch, Adler, and Scholz (1997) | Yes | ? | No | No | | NA | | No | Yes | Yes | Yes | 4 |
| Schäfer, Egloff, and Witthöft (2012) | No | Yes | Yes | Yes | | Yes | | Yes | Yes | Yes | Yes | 8 |
| Scholz, Ott, and Sarnoch (2001) | No | Yes | Yes | Yes | | No | | No | Yes | No | Yes | 5 |
| Schonecke (1995) | No | ? | No | No | | No | | No | Yes | No | Yes | 2 |
| Schröder, Gerlach, Achenbach, and Martin (2015) | Yes | Yes | Yes | No | | Yes | | No | Yes | Yes | Yes | 7 |
| Schulz et al. (2020) | No | Yes | Yes | Yes | | NA | | Yes | Yes | No | Yes | 6 |
| Weiss, Sack, Henningsen, and Pollatos (2014) | Yes | Yes | Yes | Yes | | Yes | | Yes | Yes | No | No | 7 |
| Witthöft, Bräscher, Jungmann, and Koteles (2020) | No | NA | NA | NA | | NA | | No | Yes | No | Yes | 2 |
| Count “yes” (percent) for somatic symptom disorders / illness anxiety disorders | 17 (62.96) | 12 (44.44) | 10 (37.04) | 14 (51.85) | 10 (37.04) | | 12 (44.44) | | 25 (92.59) | 9 (33.33) | 22 (81.48) |  |
| Functional syndromes | | | | | | | | | | | |  |
| Akyol, Ulus, Tander, Bilgici, and Kuru (2013) | No | Yes | Yes | Yes | | Yes | | No | Yes | No | Yes | 6 |
| Anastasopoulos et al. (1997) | No | Yes | No | Yes | | No | | No | Yes | Yes | Yes | 5 |
| Bara-Jimenez, Shelton, Sanger, and Hallett (2000) | No | Yes | No | Yes | | No | | No | No | No | No | 2 |
| Bara-Jimenez, Shelton, and Hallett (2000) | No | Yes | No | Yes | | No | | No | Yes | No | No | 3 |
| Bardal, Roeleveld, Ihlen, and Mork (2016) | Yes | Yes | Yes | Yes | | No | | No | Yes | No | No | 5 |
| Borg et al. (2018) | No | Yes | Yes | Yes | | ? | | Yes | Yes | No | Yes | 6 |
| Brun, McCabe, and Mercier (2020) | Yes | No | No | Yes | | No | | Yes | No | No | No | 3 |
| Celenay, Mete, Coban, Oskay, and Erten (2019) | No | Yes | Yes | Yes | | No | | No | Yes | No | Yes | 5 |
| Cheng, Wang, Lin, Wang, and Lin (2010) | No | Yes | No | Yes | | Yes | | No | Yes | No | No | 3 |
| Demartini et al. (2016) | Yes | Yes | Yes | Yes | | Yes | | Yes | Yes | No | No | 7 |
| Demartini et al. (2017) | Yes | Yes | No | Yes | | ? | | No | Yes | No | No | 4 |
| De Pauw et al. (2017) | No | Yes | No | Yes | | ? | | No | Yes | No | Yes | 4 |
| De Zoete, Osmotherly, Rivett, and Snodgrass (2020) | Yes | Yes | No | Yes | | Yes | | No | Yes | No | Yes | 6 |
| Dumas et al. (2001) | No | Yes | No | Yes | | No | | No | Yes | No | Yes | 4 |
| Duschek, Montoro, and del Paso (2017) | Yes | Yes | Yes | Yes | | Yes | | Yes | Yes | No | No | 7 |
| Edmondston et al. (2007) | Yes | Yes | Yes | Yes | | Yes | | No | Yes | No | No | 6 |
| Elsig et al. (2014) | No | Yes | Yes | Yes | | Yes | | No | Yes | No | Yes | 5 |
| Fiorio et al. (2007) | No | Yes | Yes | Yes | | Yes | | No | Yes | No | No | 4 |
| Fiorio et al. (2008) | No | Yes | Yes | Yes | | No | | No | No | No | No | 3 |
| Fiorio et al. (2011) | No | ? | No | No | | No | | No | Yes | No | No | 1 |
| Gajdos, Chriszto, and Rigo (2020) | Yes | NA | NA | NA | | NA | | Yes | Yes | No | No | 3 |
| Goncalves and Silva (2019) | No | Yes | No | Yes | | No | | No | Yes | No | Yes | 4 |
| Grip, Sundelin, Gerdle, and Karlsson (2007) | Yes | ? | No | No | | Yes | | No | No | No | No | 2 |
| Jungilligens et al. (2020) | No | Yes | Yes | Yes | | Yes | | Yes | Yes | No | No | 6 |
| Katschnig et al. (2010) | No | Yes | Yes | Yes | | No | | No | Yes | No | No | 4 |
| Koreki et al. (2020) | Yes | Yes | No | Yes | | No | | No | Yes | No | No | 4 |
| Kristjansson, Dall'Alba, and Jull (2003) | Yes | Yes | Yes | Yes | | Yes | | No | Yes | No | No | 6 |
| Lee, Wang, Yao, and Wang (2008) | Yes | NA | NA | NA | | NA | | No | Yes | No | Yes | 3 |
| Marinelli et al. (2011) | No | Yes | No | Yes | | No | | Yes | No | Yes | No | 4 |
| Morgante et al. (2011) | No | Yes | Yes | Yes | | No | | No | Yes | No | Yes | 5 |
| Nijs, Aerts, and De Meirleir (2006) | Yes | Yes | Yes | Yes | | Yes | | No | Yes | No | Yes | 7 |
| Pick et al. (2020) | Yes | No | Yes | Yes | | No | | Yes | Yes | No | Yes | 6 |
| Pinsault, Vuillerme, and Pavan (2008) | No | ? | No | No | | Yes | | No | Yes | No | Yes | 3 |
| Ricciardi et al. (2016) | No | Yes | Yes | Yes | | No | | Yes | Yes | No | Yes | 6 |
| Rost, Van Ryckeghem, Schulz, Crombez, and Vogele (2017) | Yes | Yes | Yes | Yes | | No | | No | Yes | No | Yes | 6 |
| Sanger, Tarsy, and Pascual-Leone (2001) | No | ? | No | No | | No | | No | Yes | No | Yes | 2 |
| Scontrini et al. (2009) | No | Yes | No | Yes | | No | | No | Yes | No | No | 3 |
| Sjolander, Michaelson, Jaric, and Djupsjobacka (2008) | No | Yes | Yes | Yes | | Yes | | No | Yes | Yes | Yes | 7 |
| Tinazzi, Frasson, Bertolasi, Fiaschi, and Aglioti (1999) | No | Yes | No | Yes | | No | | No | No | No | No | 2 |
| Tinazzi et al. (2002) | No | Yes | No | Yes | | No | | No | No | No | No | 2 |
| Ulus, Akyol, Tander, Bilgici, and Kuru (2013) | No | Yes | Yes | Yes | | No | | No | Yes | No | Yes | 5 |
| Valenzuela-Moguillansky, Reyes-Reyes, and Gaete (2017) | Yes | Yes | Yes | Yes | | No | | Yes | Yes | No | Yes | 7 |
| Woodhouse and Vasseljen (2008) | Yes | No | Yes | Yes | | Yes | | No | Yes | No | No | 5 |
| Count “yes” for functional syndromes (percent) | 16 (38.10) | 34 (80.95) | 22 (52.38) | 36 (85.71) | | 15 (35.71) | | 9 (21.43) | 33 (78.57) | 3 (7.14) | 20 (47.62) |  |
| Count “yes” for the whole sample (percent) | 33 (47.83) | 46 (66.67) | 32 (46.38) | 50 (72.46) | | 25 (36.23) | | 21 (30.43) | 58 (84.06) | 12 (17.3) | 42 (60.87) |  |
